# Widespread, bipartisan aversion exists to neighbors owning AR-15s or storing guns insecurely

**DOI:** 10.1073/pnas.2311825121

**Published:** 2024-04-08

**Authors:** Justin L. Sola, Justin T. Pickett

**Affiliations:** ^a^Department of Sociology and School of Data Science and Society, University of North Carolina-Chapel Hill, Chapel Hill, NC 27599-3210; ^b^School of Criminal Justice, University at Albany, State University of New York, Albany, NY 12222

**Keywords:** firearm ownership, social preferences, gun control, safe storage, AR-15

## Abstract

Over 45,000 gun deaths occur annually in the United States, a country with more than 100 million gun owners and more than 350 million guns. Nevertheless, passing legislation to reduce gun violence is difficult because the issue is intensely polarized. Polls asking about general gun policies (e.g., AR-15 restrictions) demonstrate that, at least in the abstract, Americans disagree vehemently about whether civilians should be able to keep and bear arms. It is possible, however, that a hidden consensus exists in America, which has thus far escaped attention—specifically, that when the focus is on their immediate environments and daily lives, even traditionally pro-gun groups may exhibit aversion to certain types of gun ownership and storage practices. To test this, we conducted two preregistered survey experiments with a large national sample. The first was a conjoint analysis where respondents chose between neighbors (n = 33,596 choices) who randomly varied on seven attributes, including gun ownership (none, pistol, AR-15). No group of respondents, not even traditionally pro-gun groups (e.g., Republicans), exhibited a significant preference for living near gun owners, and every group was averse to AR-15-owning neighbors. The second experiment, per debates about safe-storage laws, was a picture-based factorial vignette that randomized a neighbor’s gun storage practices (n = 2,098). Every group of respondents was averse to interacting with a neighbor who stored guns outside of a locked safe. Our findings demonstrate that there is widespread agreement that certain types of gun ownership and storage practices are undesirable for communities.

## Significance

Despite the epidemic of gun violence in the United States, polarization frustrates regulation efforts (e.g., AR-15 restrictions, safe-storage laws). At least in the abstract, strong disagreement exists about whether civilians should have ready access to guns. In two preregistered survey experiments, we found that there is much more consensus among Americans when the focus is on their local communities. Even traditionally pro-gun groups (e.g., Republicans, gun owners) did not want their neighbors to have ready access to guns. Instead, they exhibited significant discomfort with neighbors owning AR-15s or storing guns for quick, self-defense access (i.e., loaded and unlocked). These findings reveal that a widespread agreement exists that AR-15 ownership and insecure storage are undesirable for communities.

Expressing one’s constitutional rights does not typically threaten people’s lives. The notable exception is gun ownership (1). Gun violence, both accidental and deliberate, is a leading cause of death in the United States; it now kills more Americans than motor vehicle accidents (2). The number of mass shootings has also increased sharply, and they have become deadlier (3). The “unrelenting epidemic” of gun violence (4) resulted in over 45,000 deaths in both 2020 and 2021 (5). A stock of over 350 million civilian firearms (6), the largest in the world (7), facilitates the toll of gun suicides, injuries, and homicides. This stock has surged because Americans are purchasing firearms at an unprecedented rate—up to 2 million per month (8, 9).

Attempts to address the epidemic of gun violence face many obstacles. Emotional responses to mass shootings are localized, short-lived, and politicized (10), providing only limited (by place, time, and political party) windows of heightened support for reform (11). Gun purchasing also increases in the aftermath of mass shootings (12). Perhaps the largest obstacle to gun reform, however, is polarization (13, 14), which has increased tremendously in recent decades (15, 16). When considering policies at a general or abstract level, Americans now disagree strongly about whether additional gun regulations are needed and about what reforms would be most useful. To illustrate, although most Democrats (88%) and Independents (56%) support stricter gun laws, only 26% of Republicans do (16).

Yet, despite intense polarization in the abstract, there are reasons to believe that Americans may exhibit more agreement about guns when the focus is on their immediate environments. First, in many other policy domains, such as housing, electricity, and emergency shelters, research shows that people’s general policy attitudes frequently differ from their views about local affairs (17–19). Second, ardent gun advocacy organizations, like the National Rifle Association (20) and the National Shooting Sports Foundation (21), often restrict firearms at their events, suggesting a gulf between abstract gun attitudes and local preferences. Third, Americans overestimate others’ opposition to many types of gun regulations (22), meaning that pluralistic ignorance may sustain general policy attitudes that are inconsistent with individuals’ private preferences about their own lives and local communities (23). For these reasons, when the lens shifts to Americans’ immediate environments (e.g., neighborhoods), it is possible that abstract polarization may dissipate to reveal common ground. Individuals in traditionally pro-gun groups (e.g., Republicans, gun owners) and those who are not in such groups may both exhibit aversion to their neighbors having ready access to guns. Thus, our first question is as follows: Do pro-gun groups, and Americans in general, want to live near and interact with gun owners?

Any reservations that Americans, whether in pro-gun groups or not, have about living near gun owners are likely to be most pronounced for certain types of ownership practices, such as owning an AR-15 or storing guns insecurely. The AR-15 is now the most popular rifle in America, with between 25 and 44 million such “modern sporting rifles” in circulation as of 2022 (24). However, the rifle’s 30-round magazine, military adoption, and close association with mass shootings make it an object of fear and controversy in America (25). Notably, only a minority of Americans, and less than 1 in 4 Republicans, support banning AR-15s (26). However, do pro-gun groups, or Americans in general, feel comfortable living near people who own AR-15s?

An equally important question is the following: Do people in pro-gun groups, or do Americans in general, want to be around or interact with gun owners who store their guns for quick, self-defense access (i.e., unlocked and loaded)? Studies have found that safe storage laws are one of the most effective forms of gun regulation, decreasing accidental shootings and suicides, particularly among children (27, 28). This is important because accidental shootings involving unsecured guns and children occur with alarmingly high frequency (6). Yet, some gun advocates worry that requiring owners to lock their guns compromises public safety by delaying access to weapons needed to prevent crime (29, 30). Consequently, most gun owners keep unlocked guns in their homes (31). However, do gun owners and other pro-gun groups feel comfortable when their neighbors store guns for quick, self-defense access?

The current study answers the above questions by testing experimentally whether Americans, including those in traditionally pro-gun groups, are averse to certain types of gun ownership practices occurring in their neighborhoods. We conducted two preregistered survey experiments with a national sample of US residents. One was a conjoint analysis that tested how a potential neighbor’s gun ownership status (e.g., owns an AR-15) affected preferences for having them move nearby (study 1; n_1_ = 33,596 choices, n_2_ = 2,105 respondents). The second was a picture-based factorial vignette that tested how a neighbor’s gun storage practice affected respondents’ willingness to interact with them (study 2; n = 2,098). The experimental results reveal a nuanced view of gun possession among traditionally pro-gun groups (e.g., Republicans, gun owners). These groups do not want to live near neighbors who own AR-15s, nor do they want to be around people who store guns for quick, self-defense access. Our findings, by demonstrating that even Americans in traditionally pro-gun groups understand that certain gun ownership practices are undesirable for communities, contribute to ongoing policy discussions about the need to balance gun rights against risks of injury, homicide, and suicide.

## Materials and Methods

### Sample.

The experiments were embedded in a Qualtrics survey administered to a large (N = 2,135) national sample of adult (18+) US residents, who were recruited online through CloudResearch Connect (32). Participants were presented with study information for their review before consenting to proceed in the survey. The survey was conducted in accordance with the guidelines of the Institutional Review Board at the University of California, Irvine (Protocol #2759), where it was determined to be exempt.

Following recommendations for survey experiments, we used a purposive sampling strategy focused on ensuring range (or diversity) (33), and the resulting sample was diverse demographically, politically, socioeconomically, and geographically. Importantly, about 35% of respondents reported having a gun in their home, which closely matches the prevalence estimate from NORC’s 2022 General Social Survey (34).

The full descriptive statistics for the sample are provided in the online supplement, along with the corresponding population parameters (*SI Appendix*, Table S2). As with most online samples, ours differed from the adult US population in some respects (e.g., on age and education). Despite such differences, experimental findings from online samples like ours still exhibit strong generalizability (35, 36). Additionally, in our experiments, weighting the sample to match the population yields very similar findings, although the estimates are less precise (*SI Appendix*, Figs. S19 and S22). Per the standard practice in analyzing online survey-experimental data (35, 37–38), however, and per our preregistration (*SI Appendix*), our main models are estimated with unweighted data.

### Experiment 1.

Our first experiment, which tested whether respondents preferred gunless neighbors, was a paired-profile conjoint analysis (37, 38). We presented each respondent with sixteen potential neighbors (paired in eight conjoint tables) and asked them to choose which (in each pairing) they would prefer to have living in their neighborhood. The potential neighbors were defined by seven attributes with randomized levels, amounting to a 3^5^4^2^ factorial design. The attribute of interest was the potential neighbor’s gun ownership. It had three levels: not an owner, owns a pistol, or owns an AR-15. The other six randomized attributes were factors that respondents might assume to be correlated with gun ownership: the potential neighbor’s gender, race/ethnicity, political party, religious affiliation, family status, and socioeconomic status. We included these “control” attributes to minimize construct confounding (39) and to increase realism, by ensuring choices were multifaceted and involved trade-offs (40). We randomized the order of attributes for each participant—holding it constant across their conjoint tables—and randomized the levels of each attribute in each profile (37).

After listwise deletion of missing data (<2% of cases), the first experiment resulted in 33,596 choice outcomes clustered in 2,105 respondents. This analytic sample size (and experimental design) yielded over 90% statistical power to detect an average marginal component effect (AMCE) of 0.05 for the potential neighbor’s gun ownership, both in the full-sample analysis and in each of the subsample analyses. The standard practice for forced-choice conjoint experiments is to estimate AMCEs using ordinary least squares (OLS) regression with data at the profile level and SE clustered at the respondent level (37, 38). Per our preregistration, that is how we analyzed the data.

### Experiment 2.

The second experiment tested how a neighbor’s gun storage practice affected respondents’ willingness to interact with them. Here, we used a randomized vignette with an appended picture and a 2^4^3^1^ factorial design (40). Specifically, we asked respondents to imagine attending a get-together at a new neighbor’s home, during which a drawer is opened revealing a stored pistol (Table 1). Along with the vignette text, respondents received a picture of the drawer and its contents, wherein the type of gun storage was randomized: 50% secure (locked and unloaded) and 50% insecure (unlocked and loaded). Because two main types of secure storage exist, we used both: 25% gun safe and 25% chamber lock (Table 1). To ensure realism and information equivalence (39, 40), we also randomized the attributes of the neighbor (family type, temperament, alcohol consumption) and of the get-together (enjoyability). Respondents then answered two Likert scale questions about their likelihood (0 = very unlikely, 4 = very likely) of interacting with that neighbor in the future, either at the neighbor’s home or at their own home (Table 1). Per our preregistration, we averaged the responses to these two questions (*r* = 0.79, α = 0.88) to generate the measure of social interaction preferences.

**Table 1.** Second experiment: Factorial vignette

Vignette Text
Imagine that [Manipulation A] moves into YOUR NEIGHBORHOOD. Shortly after they move in, they invite you over one evening to a get-together at their home with some of their friends. You go and it is your first time at their home. [Manipulation B]. While there, someone opens a drawer and [Manipulation C]. Over the course of the night, [Manipulation D].
Manipulation A: Neighbor’s Family Type
1) a young couple without children
2) a young couple with children
Manipulation B: Neighbor’s Temperament and Drinking
1) The couple seems happy.
2) The couple has several beers and seems happy.
3) The couple argues a few times.
4) The couple has several beers and argues a few times.
Manipulation C: Gun Storage
1) you notice a pistol, a loaded magazine, and some ammunition in the drawer, pictured below. One of the owners tells you it is a loaded 9 mm pistol that they keep there in case they ever need it for protection. [Picture A]
2) you notice a pistol with a chamber lock, a loaded magazine, and some ammunition in the drawer, pictured below. One of the owners tells you it is a locked 9 mm pistol that they keep there in case they ever need it for protection. [Picture B]
3) you notice a locked pistol safe in the drawer, pictured below. One of the owners tells you it contains a loaded 9 mm pistol that they keep locked inside in case they ever need it for protection. [Picture C]
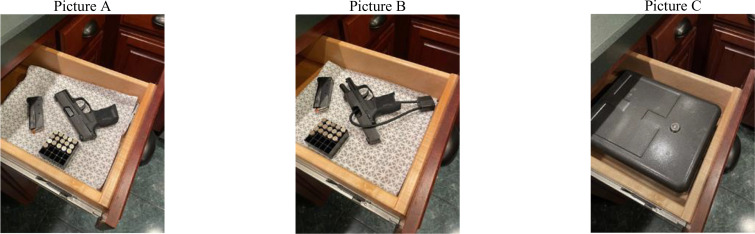
Manipulation D: Enjoyability of Experience
1) you have an okay time.
2) you have a good time.
Follow-Up Questions
How likely or unlikely is it that, if invited back again, you would go to another get-together at their house in the future? (Very likely, likely, neither likely nor unlikely, unlikely, very unlikely)
How about on your end? How likely or unlikely is it that you would invite this couple over to your house for a get-together? (Very likely, likely, neither likely nor unlikely, unlikely, very unlikely)

In the second experiment, after listwise deletion of missing data (<2% of cases), the total analytic sample size was 2,098 respondents. This sample size, with the experimental design, yielded over 90% statistical power to detect medium-sized effects of the neighbor’s gun storage practice in both the full-sample and subsample analyses. Per the preregistration, and given the distribution of the outcome variable (a multiple-item index), we estimated the models using OLS regression with robust SE.

### Compliance Checks.

Two compliance checks were included in the survey. The first repeated the first conjoint table later in the survey with the profile (column) order swapped, so that respondents had to invert their earlier choice to choose the same neighbor (41). Most (86%) respondents did so. The second check was a screener that appeared to be a normal question, but included hidden instructions at the end of a lengthy question stem (42). Those instructions were the following: “to show you are reading, please just skip to the next page without answering this question.” As instructed, almost all respondents (96%) skipped the question. Overall, 82% of respondents passed both checks. Per the preregistration, in both experiments, we first estimated the intent-to-treat (ITT) effects, using all respondents, after which we dropped noncompliers and estimated the average effects of treatment receipt for compliers (AERC) (43).

### Pro-Gun Subsamples.

Past research shows that Republicans, gun owners, and those socialized as children into gun culture tend to have more favorable attitudes toward guns and are more likely to oppose gun control (44–48). Another pro-gun group is gun desirers (i.e., people who want guns) (9). We therefore estimated subsample models by partisan identification (non-Republican vs. Republican), by household gun ownership (non-owners vs. owners), by childhood gun socialization (below vs. above the mean on a five-item index, α = 0.81), and by gun desirability (below vs. above the mean on a three-item index, α = 0.85). The questions used to measure these respondent characteristics were included in the survey after the two experiments. The exact question wording is provided in the *SI Appendix*.

## Results

### Experiment 1: Preferences for Gunless Neighbors.

This experiment answered three questions: 1) Do people want to live near gun owners? 2) Do members of pro-gun groups (e.g., Republicans, gun owners) want to live near gun owners? 3) Does the type of gun (e.g., AR-15) owned by a potential neighbor matter? These questions were answered using a conjoint experiment (37, 38), wherein respondents evaluated eight pairs of potential neighbors and chose which (in each pair) they preferred to live nearby. The potential neighbors’ attributes, including their gun ownership, were randomized.

**Fig. 1.**
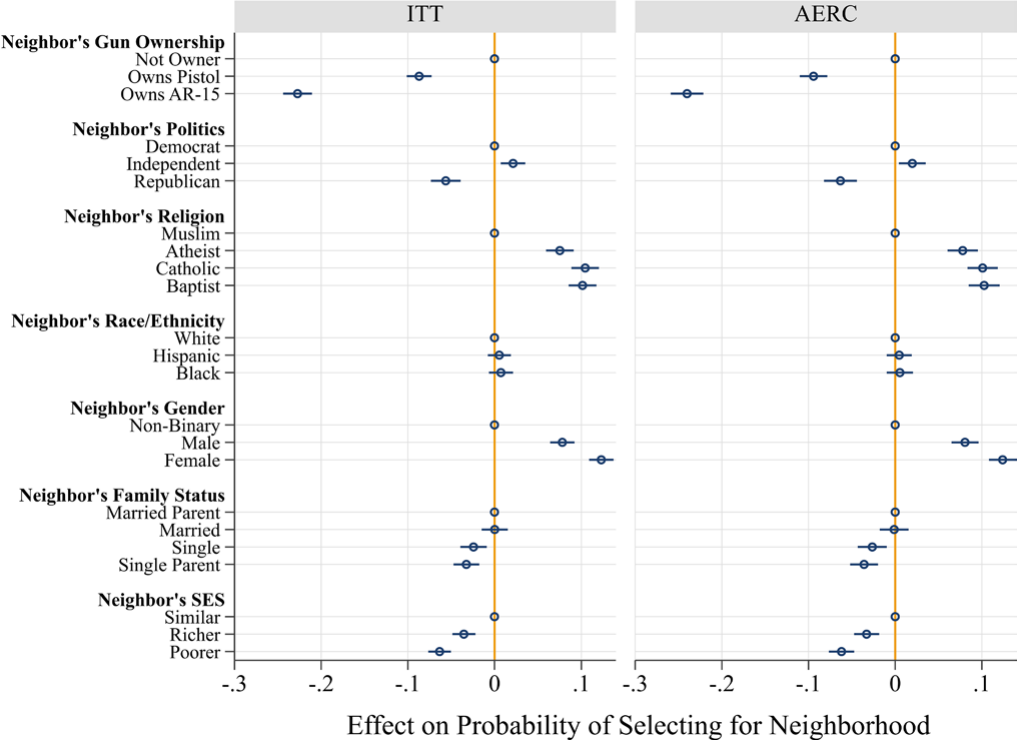
Experiment 1: Effects of potential neighbor’s attributes on selection for neighborhood. Models are estimated using linear regression with robust SE clustered at the respondent level. Coefficients (with 95% CI) are shown. ITT = intent-to-treat effect, AERC = average effect of receipt of treatment for compliers. The sample sizes are: 33,596 profile choices clustered in 2,105 respondents (ITT), and 27,632 profile choices clustered in 1,727 respondents (AERC).

Fig. 1 shows the AMCE in the full sample. It presents the effects for all respondents who answered (ITT) as well as those among respondents who passed the compliance checks (AERC). The findings are clear: Respondents did not want to live near gun owners. If the potential neighbor owned a pistol, the probability that respondents would choose to live near them dropped by nine percentage points (ITT: *b* = −0.087, *P* < 0.001; AERC: *b* = −0.094, *P* < 0.001). The effect of AR-15 ownership was even larger: if the potential neighbor owned an AR-15, the probability that respondents would choose to live near them plummeted by over 20 percentage points (ITT: *b* = −0.227, *P* < 0.001; AERC: *b* = −0.240, *P* < 0.001). Although most of the other randomized attributes also exerted significant effects—respondents preferred politically independent, non-Muslim neighbors who were female, married, and of a similar socioeconomic status—none mattered as much as the potential neighbor’s AR-15 ownership. Stated differently, the single best predictor of respondents’ aversion to having someone as a neighbor was if that person owned an AR-15.

**Fig. 2.**
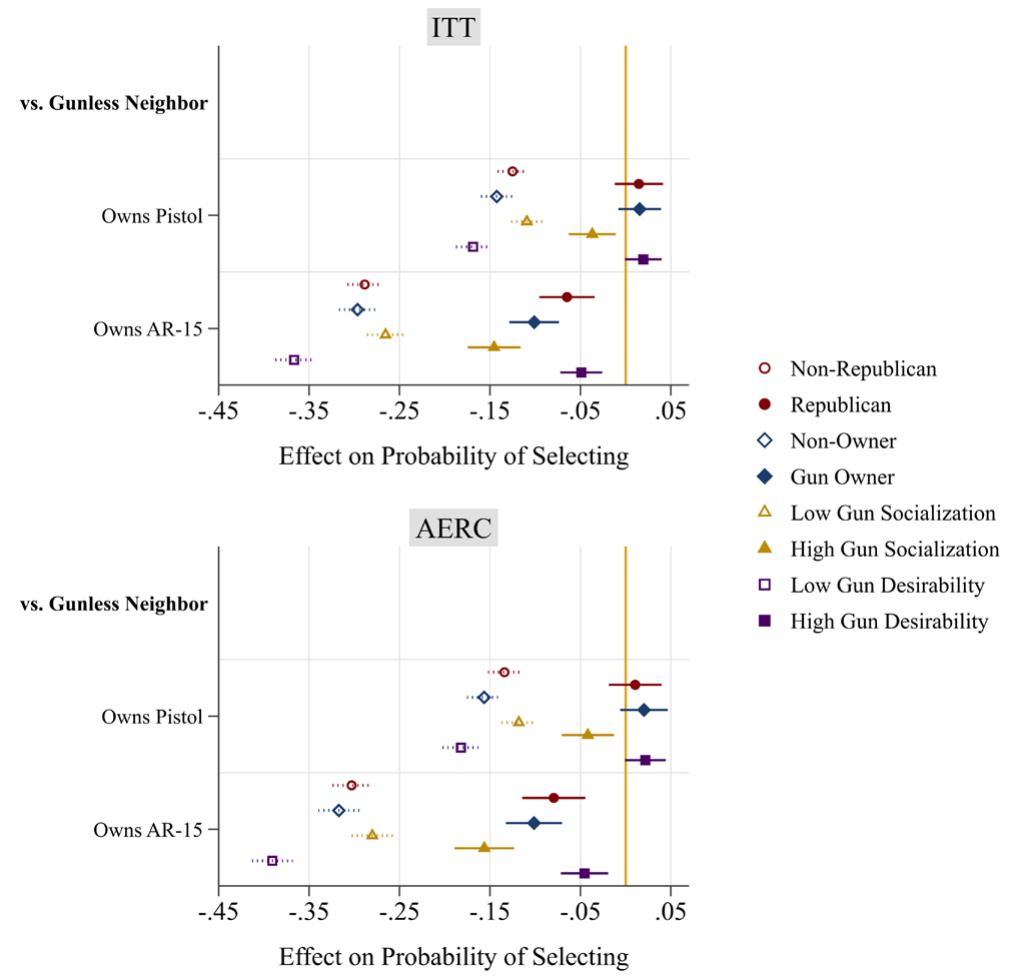
Experiment 1, Disaggregated analyses: Effects of potential neighbor’s gun ownership on selection for neighborhood. Models are estimated using linear regression with robust SE clustered at the respondent level, and control for the six other randomized attributes of the applicant. Regression coefficients (with 95% CI) are shown. “Low” is defined as at or below the mean on the variable, and “High” is above the mean. ITT = intent-to-treat effect, AERC = average effect of receipt of treatment for compliers.

Do pro-gun groups want to live near gun owners? The findings in Fig. 2 answer this question. These findings are estimated after disaggregating the sample into traditionally pro-gun groups—Republicans, gun owners, gun desirers, and the gun socialized—vs. their counterparts. There was not a single group that exhibited a significant preference for living near gun owners, and every group was uncomfortable with AR-15-owning neighbors. First, for the choice between a pistol-owning neighbor and a gunless neighbor, members of pro-gun groups were mostly indifferent: no significant preference emerged among Republicans, among gun owners, or among gun desirers. However, gun-socialized respondents did have a preference: They preferred not to live near pistol owners (ITT: *b* = −0.037, *P* = 0.006; AERC: *b* = −0.042, *P* = 0.005).

Second, and most striking, a potential neighbor’s AR-15 ownership had a sizable, significant, and negative effect in all subgroups. Even in traditionally pro-gun groups, respondents were about 5 to 16 percentage points less likely to choose a neighbor with an AR-15, when the alternative was a gunless neighbor. Republicans preferred not to live near a neighbor with an AR-15 (ITT: *b* = −0.065, *P* < 0.001; AERC: *b* = −0.080, *P* < 0.001); gun owners preferred not to live near a neighbor with an AR-15 (ITT: *b* = −0.101, *P* < 0.001; AERC: *b* = −0.101, *P* < 0.001); gun desirers preferred not to live near a neighbor with an AR-15 (ITT: *b* = −0.049, *P* < 0.001; AERC: *b* = −0.046, *P* = 0.001); and the gun socialized preferred not to live near a neighbor with an AR-15 (ITT: *b* = −0.145, *P* < 0.001; AERC: *b* = −0.156, *P* < 0.001).

### Experiment 2: Preferences for Secure Gun Storage.

Our first experiment documented a general aversion to gun-owning neighbors. But what about when neighbors do own guns? Our second experiment dealt with this situation and focused on gun storage practices. It answered three questions: 1) Do people want to interact with gun-owning neighbors who practice insecure storage? 2) Do members of pro-gun groups (e.g., Republicans, gun owners) want to interact with gun-owning neighbors who practice insecure storage? 3) When guns are stored securely, does the storage type (e.g., gun safe, chamber lock) matter? These questions were answered using a picture-based factorial vignette (40), wherein respondents considered a hypothetical, gun-owning neighbor who used a particular storage practice (gun safe, chamber lock, or unlocked and loaded), and then indicated their likelihood of interacting with that neighbor again in the future.

**Fig. 3.**
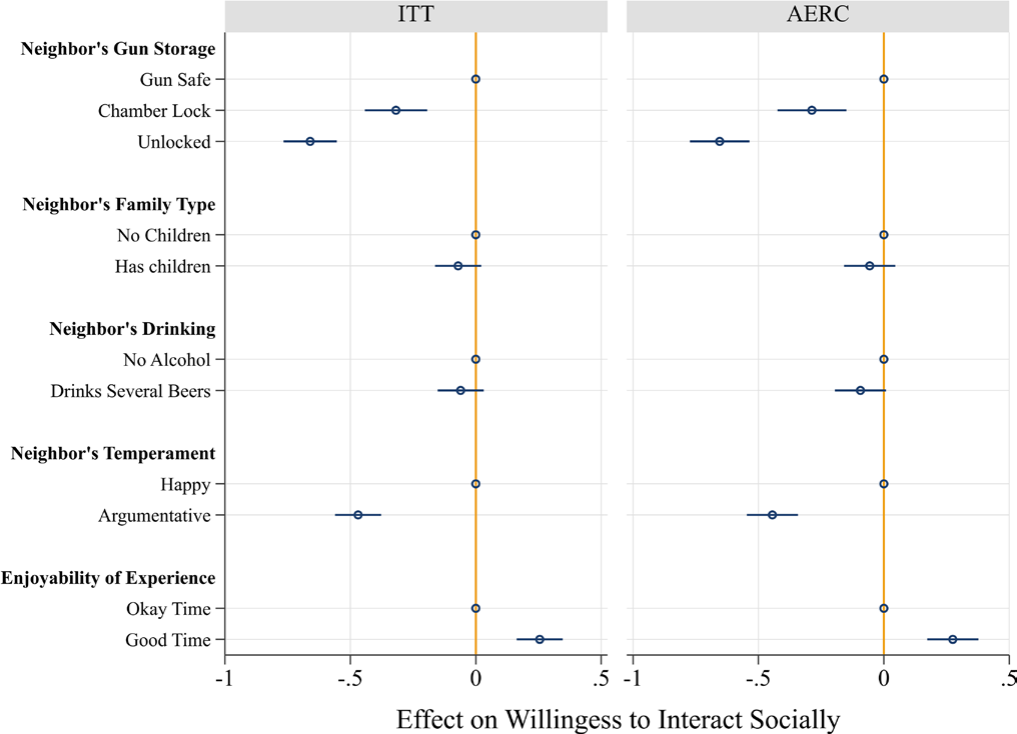
Experiment 2: Effects of neighbor’s attributes on willingness to interact socially. Models are estimated using linear regression with robust SE. Coefficients (with 95% CI) are shown. ITT = intent-to-treat effect, AERC = average effect of receipt of treatment for compliers. The sample sizes are 2,098 respondents (ITT), and 1,727 respondents (AERC).

Fig. 3 shows the effects in the full sample. Gun storage practices mattered greatly for social interaction preferences. Respondents were significantly less willing to interact with a neighbor who stored a gun unlocked (ITT: *b* = −0.660, *P* < 0.001; AERC: *b* = −0.654, *P* < 0.001) or with a chamber lock (ITT: *b* = −0.318, *P* < 0.001; AERC: *b* = −0.287, *P* < 0.001), compared to one who used a gun safe. Two other vignette factors also mattered: respondents were less willing to interact with a neighbor who argued with their spouse or who was less enjoyable to be around. The neighbor’s gun storage practice, however, was the single most important factor for predicting respondents’ willingness to interact with them. The absolute impact of insecure storage (unlocked and loaded) was over 40% larger than that of the neighbor’s temperament and over 130% larger than that of interaction quality.

**Fig. 4.**
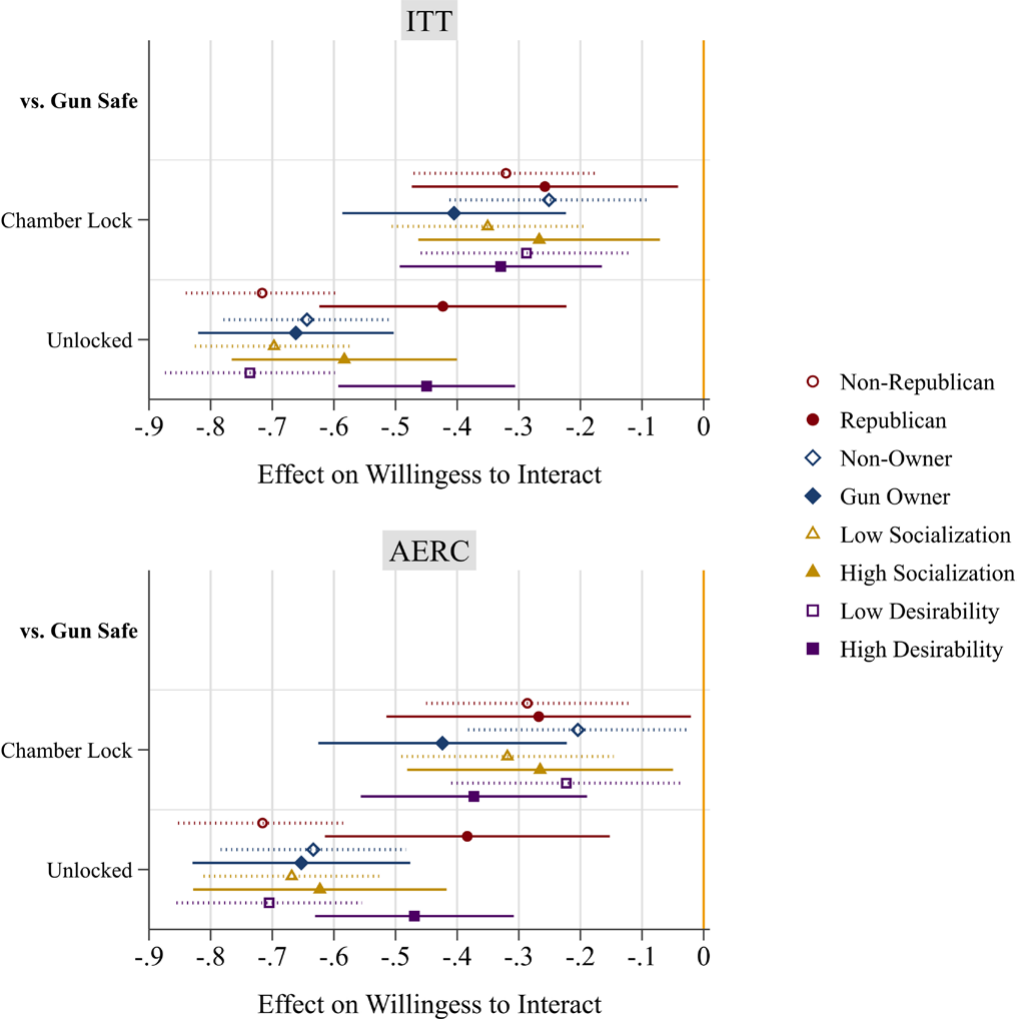
Experiment 2: Disaggregated analyses: Effects neighbor’s gun storage on willingness to interact socially. Models are estimated using linear regression with robust SE. Coefficients (with 95% CI) are shown. “Low” is defined as at or below the mean on the variable and “High” is above the mean. ITT = intent-to-treat effect, AERC = average effect of receipt of treatment for compliers.

Does insecure gun storage also deter interaction among pro-gun groups? The findings in Fig. 4 reveal that the answer to this question is yes. Fig. 4 shows the causal effects of a neighbor’s gun storage on respondents’ willingness to interact with them, separating traditionally pro-gun groups—Republicans, gun owners, gun desirers, and the gun socialized—and their counterparts. Every group of respondents was averse to interacting with a neighbor who stored guns outside of a locked safe. Republicans were less willing to interact with a neighbor who stored a gun unlocked (ITT: *b* = −0.423, *P* < 0.001; AERC: *b* = −0.383, *P* = 0.001) or who used only a chamber lock (ITT: *b* = −0.258, *P* = 0.019; AERC: *b* = −0.268, *P* = 0.034). Similarly, gun owners were less willing to interact with a neighbor who stored a gun unlocked (ITT: *b* = −0.662, *P* < 0.001; AERC: *b* = −0.653, *P* < 0.001) or who used only a chamber lock (ITT: *b* = −0.405, *P* < 0.001; AERC: *b* = −0.424, *P* < .001). Gun desirers were also less willing to interact with a neighbor who stored a gun unlocked (ITT: *b* = −0.449, *P* < 0.001; AERC: *b* = −0.469, *P* < 0.001) or who used only a chamber lock (ITT: b = −0.329, *P* < 0.001; AERC: *b* = −0.373, *P* < 0.001). The same story emerged for the gun socialized, who were less willing to interact with a neighbor who stored a gun unlocked (ITT: *b* = −0.583, *P* < 0.001; AERC: *b* = −0.623, *P* < .001) or who used only a chamber lock (ITT: *b* = −0.267, *P* = 0.008; AERC: *b* = −0.265, *P* = 0.016).

## Discussion

The gun debate in the United States is increasingly polarized (14–15), juxtaposing pro-gun beliefs in the importance of the Second Amendment and perceptions of the beneficial effects of gun ownership against the well-documented physical, emotional, community, and economic costs of gun violence (1). The CDC estimates the financial burden of firearm-related deaths was $483.9 billion dollars in 2020 (49). Incorporated into this statistic is the value of lives cut short. Recent tragedies are illustrative: After a family asked their neighbor to stop shooting an AR-15 so that their baby could sleep, the neighbor massacred the family, killing five of them, including their 9-y-old son (50); when a 2-y-old boy found an unsecured pistol, he accidently killed his pregnant mother and unborn sibling (51). In the United States, accidental shootings involving children and insecurely stored guns now occur almost every other day (6).

According to gun advocacy organizations, such as the National Rifle Association, and according to the Republican Party, it benefits society when civilians keep and bear arms, because guns enable “Americans to exercise their God-given right of self-defense for the safety of their homes, their loved ones, and their communities” (52). Correspondingly, members of pro-gun groups are much less likely than other Americans to support, at least in abstract, laws restricting or regulating gun ownership. Among Republicans, for example, only a small minority (<30%) support stricter gun control laws in general or banning AR-15 sales specifically (16, 26). The relatively low levels of support for gun control among Americans in pro-gun groups (e.g., among Republicans, gun owners), and the corresponding polarization of gun politics (14–15), make it difficult to pass gun restrictions at the national level.

What our experiments demonstrate, however, is that the abstract polarization that impedes attempts at the national level to reduce gun violence dissipates when the focus is on individuals’ immediate environments and daily lives. Even members of traditionally pro-gun groups (e.g., Republicans, gun owners) are averse to their neighbors owning AR-15s and to interacting with neighbors who store guns for quick, self-defense access (unlocked and loaded). As would be expected, other Americans (e.g., non-Republicans, non-owners) share these aversions. A consensus thus appears to exist in this country that certain types of gun ownership and storage practices are undesirable for communities: Americans, including those in pro-gun groups, do not want their neighbors to own AR-15s or to store guns insecurely.

A growing body of literature finds that common ground can be a basis for prosocial behavior changes (53). Therefore, our findings point to a potential path forward in the gun debate. They reveal that Americans widely recognize that certain gun practices do not belong in neighborhoods. For this reason, it may be possible to mitigate abstract polarization by centering local communities, and by drawing attention to the shared, bipartisan discomfort with AR-15 ownership and insecure gun storage. Individuals in pro-gun groups (e.g., Republicans), for example, may be surprised to learn that people like them (e.g., other Republicans) do not want to live near AR-15 owners or insecure gun storers. Additionally, although the Second Amendment protects the right to keep and bear arms, it does not guarantee the right to do so secretly. Consideration should thus be given to the fact that current gun policy often leaves Americans in the dark as to whether AR-15s or insecurely stored guns are next door.

Although our experiments show that a widespread aversion exists to being near people who own AR-15s or who store guns insecurely, they do not reveal why it exists. There are at least two possible explanations: Americans may widely perceive such gun practices as unsound, and/or they may look negatively upon the types of people who would engage in them. In supplementary models (*SI Appendix*, Figs. S1–S18), we explored whether respondents’ aversion to AR-15 owners and to insecure gun storers emerged regardless of the hypothetical neighbor’s other characteristics (e.g., race, religion, or party affiliation in experiment 1; social or drinking behavior in experiment 2). Respondents were always averse to these gun practices, regardless of the neighbor’s characteristics, and the effects were nearly always statistically significant. This unconditional aversion suggests the possibility of a widespread understanding that certain gun practices are dangerous no matter who engages in them. Nevertheless, future research is needed that examines the mechanisms giving rise to this aversion, perhaps by measuring perceived danger, and that explores whether there are any circumstances in which Americans are comfortable with their neighbors owning AR-15s or storing guns insecurely. It may be that few such circumstances exist.

We close by emphasizing the need for frank policy discussion on both fronts: AR-15s and gun storage. Members of pro-gun groups, just like other Americans, recognize that neither AR-15s nor insecurely stored guns are desirable in their communities. This shared sentiment should serve as the basis for effective, evidence-based policies that balance the right to bear arms with its impacts on our communities and the need for public safety. In much of America, AR-15s are as easy to buy as hunting rifles. Similarly, most gun owners are insecure storers (31). Among gun-owning parents, only a minority store all their guns locked and unloaded (54), which is problematic given that many children report knowing where to find (or even handling) their parents’ guns (55). Strong evidence exists that safe-storage laws reduce firearm-related injuries and deaths (28). Additionally, insecure storage practices contribute to the theft of approximately 400,000 guns each year (56), which are then sold and resold on the black market to facilitate violent crimes (57). Added to the evidence herein of a broad American consensus that insecurely stored guns are troubling and should be avoided, there seems to be little justification for failing to enact safe-storage laws. We have known since the 17th century that desire for weapons is itself “no sin” (58); the sin lies in our failure to implement gun policies that protect the welfare of our neighbors and communities.

## Supplementary Material

Appendix 01 (PDF)

## Data Availability

Anonymized data, code, and preregistration are available at the Open Science Foundation (osf.io/dvz6y/) (59, 60).
