# Supplementary material for: Widespread, bipartisan aversion exists to neighbors owning AR-15s or storing guns insecurely
Source: Proc Natl Acad Sci U S A. 2024 Apr 8;121(16):e2311825121. doi: 10.1073/pnas.2311825121 (PMC11032460; doi:10.1073/pnas.2311825121)
Supplement: Supplementary file 1 — Appendix 01 (PDF) [file pnas.2311825121.sapp.pdf]

Supplementary Materials for

Widespread, bipartisan aversion exists to neighbors owning AR-15s or storing guns insecurely

This file includes:

1) Preregistration, Anonymized Data, and Replication Link (p. 1)
2) First Experiment: Procedures and Question Wording (pp. 2-3)
3) Measurement of Pro-Gun Groups (pp. 4-6).
4) Analyses Disaggregated by Neighbor's Non-Gun Attributes (pp. 7-24)
    - First Experiment (pp. 7-15)
    - Second Experiment (pp. 16-24)
5) Analyses Using Sampling Weights (pp. 25-28)
    - First Experiment (pp. 25-26)
    - Second Experiment (pp. 27-28)
6) Descriptive Statistics (p. 29)

PREREGISTRATION, ANONYMIZED DATA, AND REPLICATION LINK

Available at https://osf.io/dvz6y/

FIRST EXPERIMENT: PROCEDURES AND QUESTION WORDING

Introductory Text:

On each of the next few pages, we describe two individuals. Please imagine that they are thinking of MOVING INTO YOUR NEIGHBORHOOD. Please read the descriptions carefully. Then, indicate which individual you would PREFER to have as a NEIGHBOR.

Conjoint Table Template (Attribute Ordering Randomized):

	**Neighbor A**	**Neighbor B**
**Race**		
**Gun Ownership**		
**Gender**		
**Wealth**		
**Family Status**		
**Political Party**		
**Religious Affiliation**		

Measure of Choice Outcome:

Which of these individuals would you prefer to have as a NEIGHBOR?

- o Neighbor A
- o Neighbor B

Table S1. First Experiment: Factorial Dimensions, Levels, and Text Phrases

Dimension (Attribute)	Levels and Text
Race	1) White<br>2) Hispanic<br>3) Black
Gun Ownership	1) No, not an owner<br>2) Yes, owns a pistol<br>3) Yes, owns an AR-15 rifle
Gender	1) Non-binary<br>2) Male<br>3) Female
Wealth	1) Similar to you<br>2) Richer than you<br>3) Poorer than you
Family Status	1) Married with children<br>2) Married<br>3) Single<br>4) Single with children
Political Party	1) Democrat<br>2) Independent<br>3) Republican
Religious Affiliation	1) Muslim<br>2) None, Atheist<br>3) Christian, Catholic<br>4) Christian, Baptist

NOTES: The levels of each dimension were randomized independently for each profile in each table for each participant. The ordering of the attributes within the conjoint tables was randomized between participants but held constant within participants across tables.

MEASUREMENT OF PRO-GUN GROUPS

Republican:

Question Stem:

In terms of political parties, how would you describe yourself?

Response Scale:

Strong Democrat, Democrat, Independent or Other, Republican, Strong Republican.

Coding:

We recoded responses into a binary indicator (0 = Strong Democrat, Democrat, Independent or other; 1 = Republican or Strong Republican).

Gun Ownership:

Question Stem:

Listed below are several types of firearms. Please indicate how many of each you CURRENTLY have in your home (owned by you or another household member)?

Items:
1. Semiautomatic rifle.
2. Handgun (pistol or revolver).
3. Shotgun.
4. Bolt-action rifle.

Response Scale:

None, One, Two, Three, Four or More.

Coding:

We used the response to the four items to generate a binary indicator of ownership (0 = answered "None" to all four items, 1 = owned at least one type of firearm.)

Childhood Gun Socialization:

Question Stem:

Now, please think about your CHILDHOOD (before the age of 18). Did any of your family members (or guardians) do the following things when you were growing up?

Items:

1. Take you to a gun show.
2. Teach you how to shoot a firearm.
3. Teach you how to clean a firearm.
4. Take you hunting.
5. Keep a firearm in the house.

Response Scale:

No, they did not; Yes, they did.

Coding:

We averaged responses to create an index (factor loadings: .54 to .81; $\alpha = .81$), and then divided it at the mean to create the binary indicator of low vs. high gun socialization.

Gun Desirability:

Question Stem: Take a look at the gun below: Use the slider to show how desirable this gun is to you:

Items:

1. 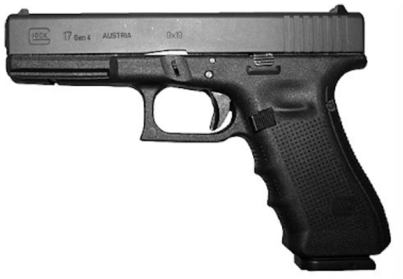

2. 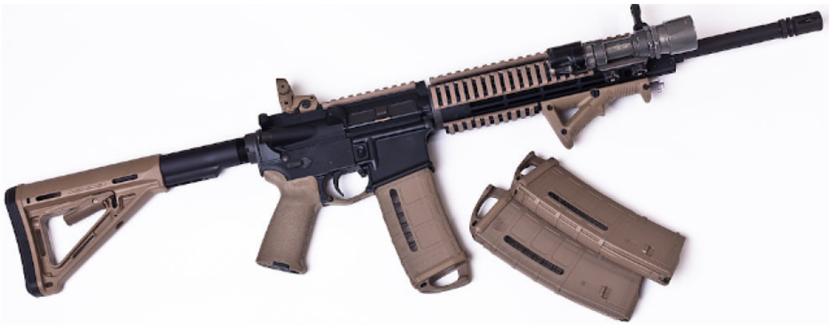

3. 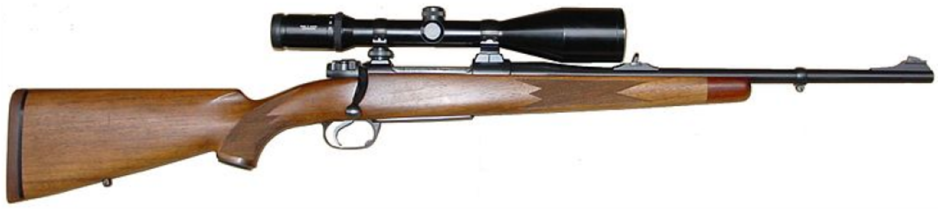

Response Scale:

Horizontal sliding scale labeled “No desire” (left bound, internally coded to 0) to “Most desire” (right bound, internally coded to 100).

Coding:

We averaged responses to create an index (factor loadings: .78 to .81; $\alpha = .85$), and then divided it at the mean to create the binary indicator of low vs. high gun desirability.

ANALYSES DISAGGREGATED BY NEIGHBOR'S NON-GUN ATTRIBUTES

- NOTE: These supplementary analyses examine the effect of a potential neighbor's gun ownership separately for each type of potential neighbor (disaggregated subsamples).

First Experiment:

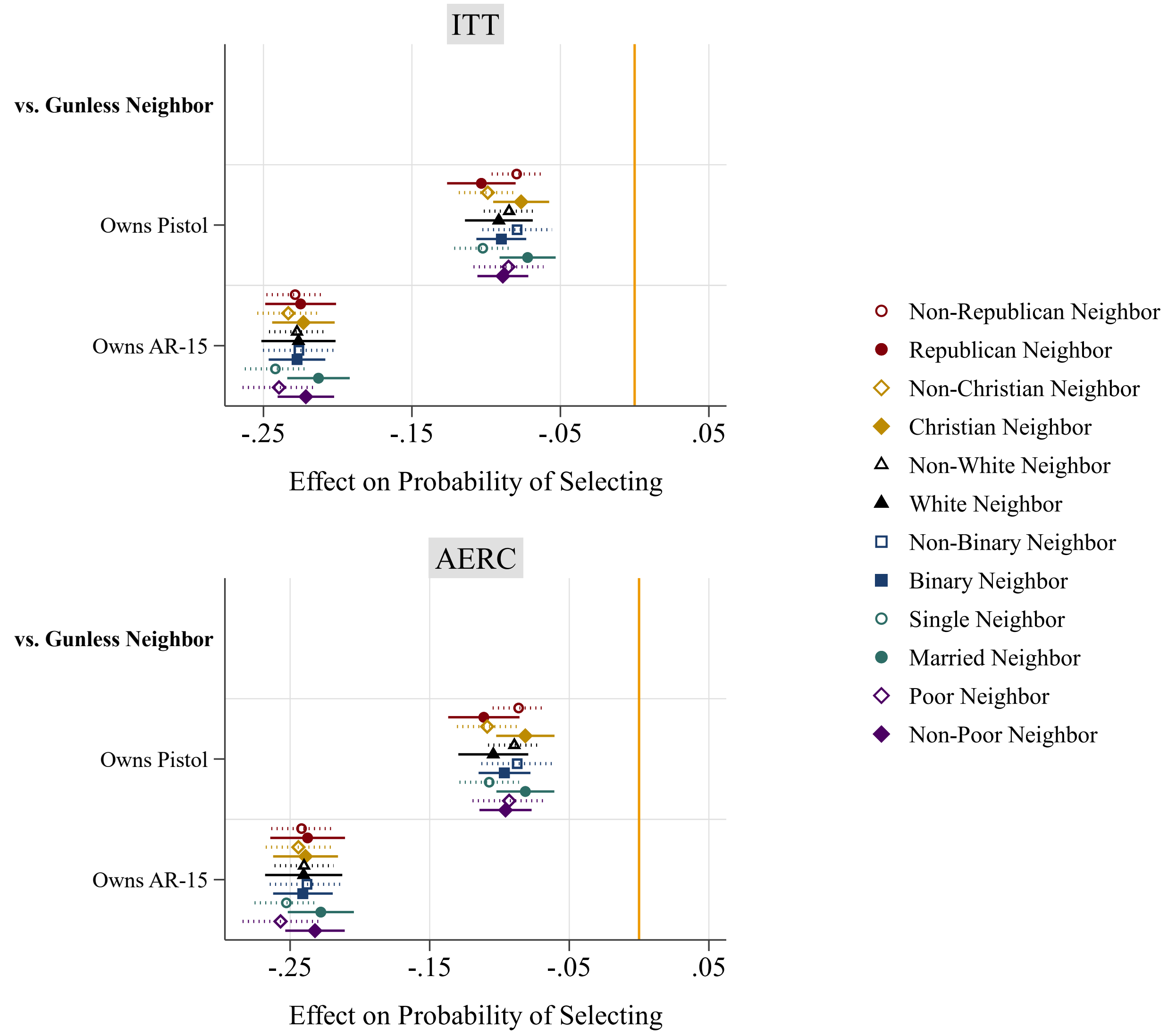

Fig. S1. Experiment 1, All Respondents: By Potential Neighbor's Attributes. This figure shows the effects of a potential neighbor's gun ownership, conditional on the neighbor's other attributes. The models are estimated using linear regression with robust standard errors clustered at the respondent level. Coefficients (with 95% confidence intervals) are shown. All models control for the other randomized attributes of the neighbor.

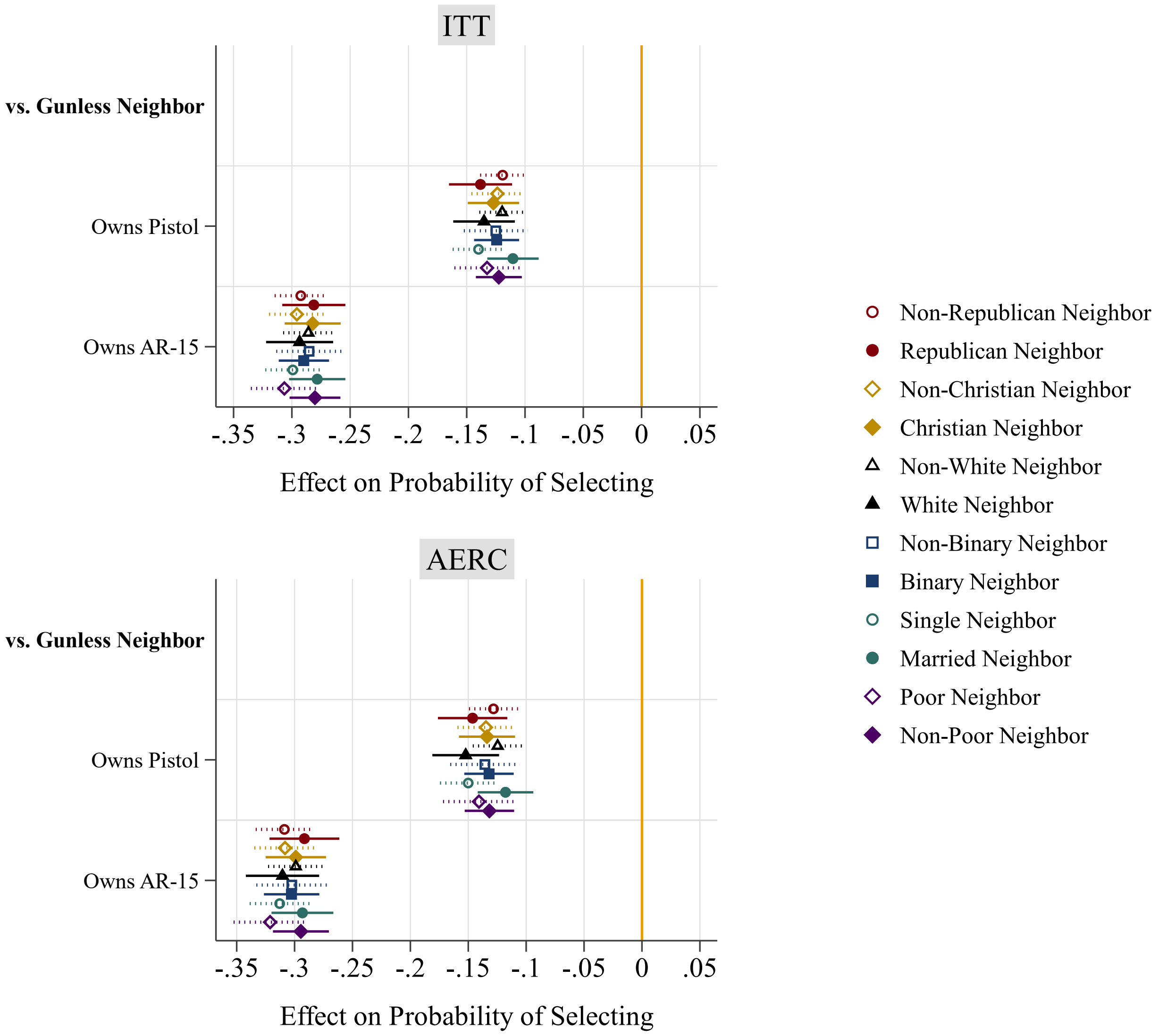

Fig. S2. Experiment 1, Non-Republican Respondents: By Potential Neighbor's Attributes. This figure shows the effects of a potential neighbor's gun ownership, conditional on the neighbor's other attributes. The models are estimated using linear regression with robust standard errors clustered at the respondent level. Coefficients (with 95% confidence intervals) are shown. All models control for the other randomized attributes of the neighbor.

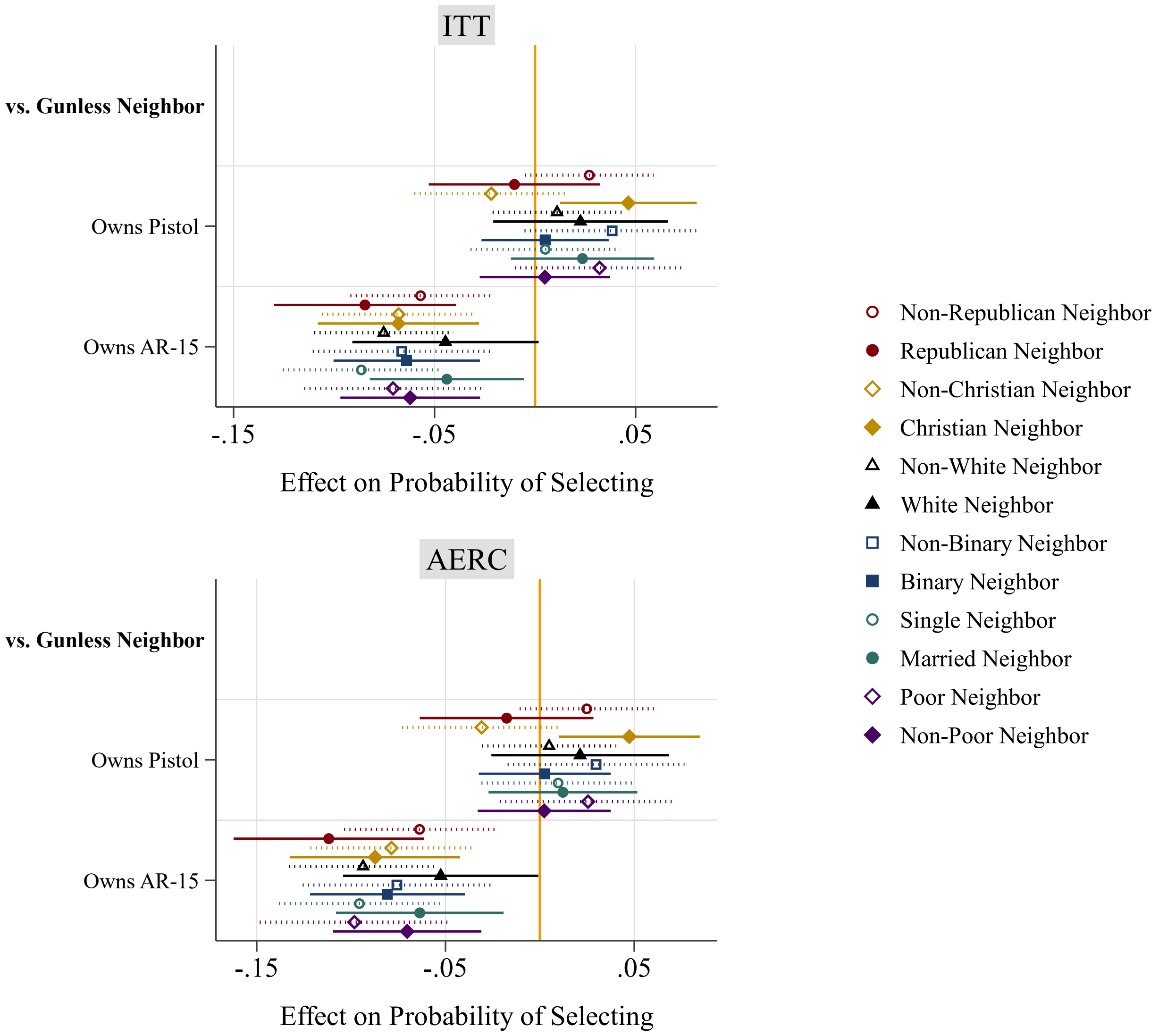

Fig. S3. Experiment 1, Republican Respondents: By Potential Neighbor's Attributes. This figure shows the effects of a potential neighbor's gun ownership, conditional on the neighbor's other attributes. The models are estimated using linear regression with robust standard errors clustered at the respondent level. Coefficients (with 95% confidence intervals) are shown. All models control for the other randomized attributes of the neighbor.

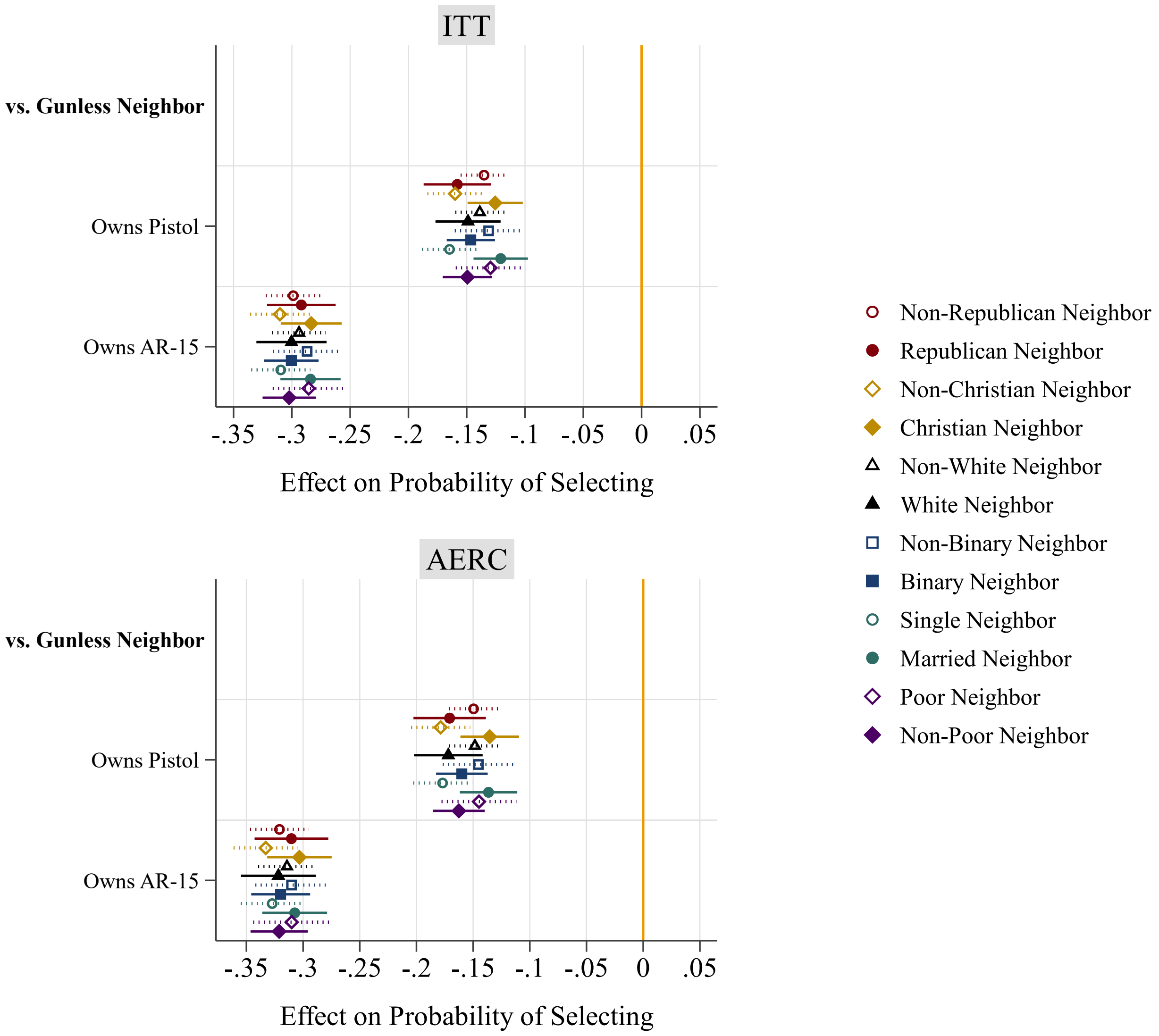

Fig. S4. Experiment 1, Non-Owner Respondents: By Potential Neighbor's Attributes. This figure shows the effects of a potential neighbor's gun ownership, conditional on the neighbor's other attributes. The models are estimated using linear regression with robust standard errors clustered at the respondent level. Coefficients (with 95% confidence intervals) are shown. All models control for the other randomized attributes of the neighbor.

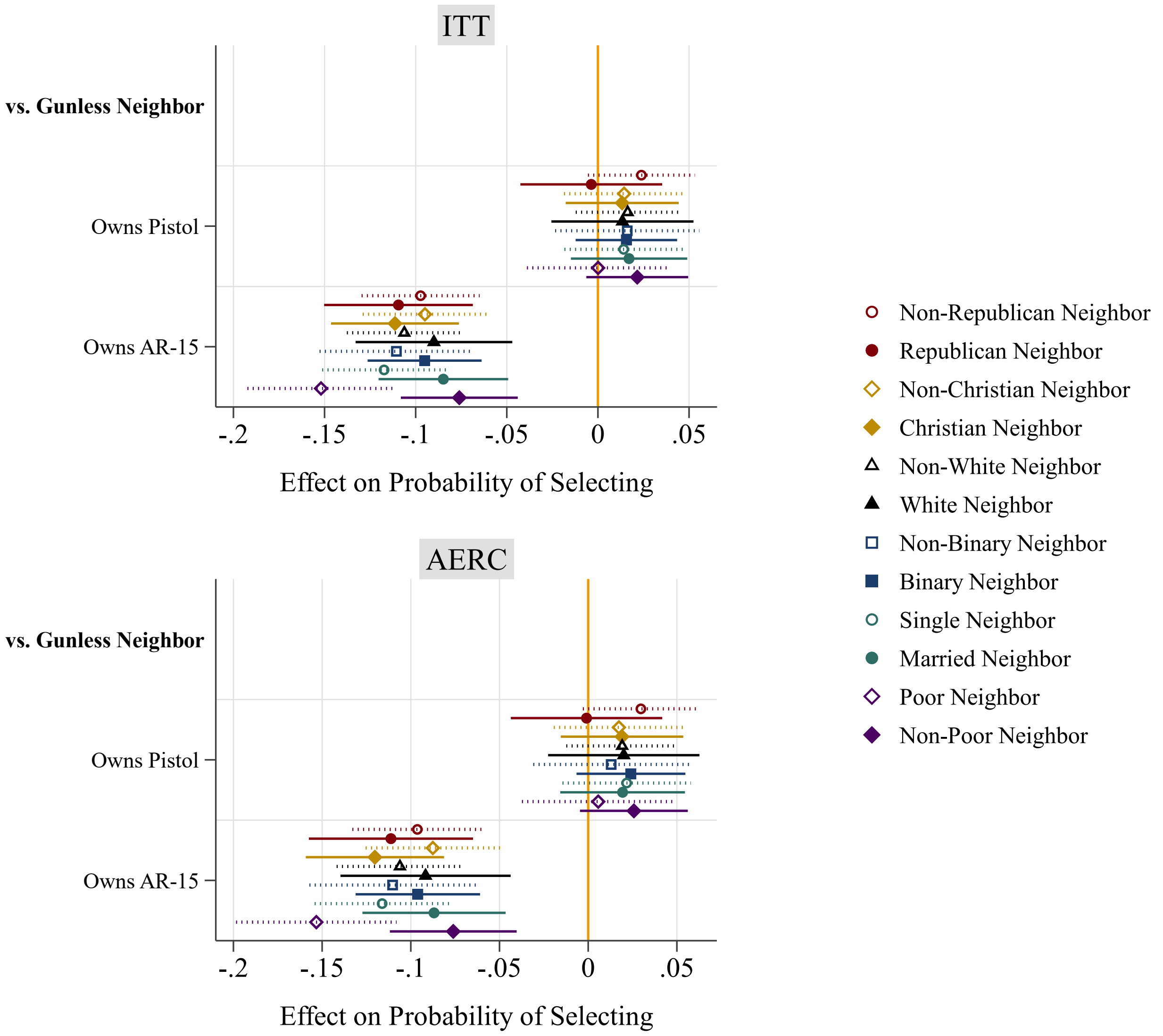

Fig. S5. Experiment 1, Owner Respondents: By Potential Neighbor's Attributes. This figure shows the effects of a potential neighbor's gun ownership, conditional on the neighbor's other attributes. The models are estimated using linear regression with robust standard errors clustered at the respondent level. Coefficients (with 95% confidence intervals) are shown. All models control for the other randomized attributes of the neighbor.

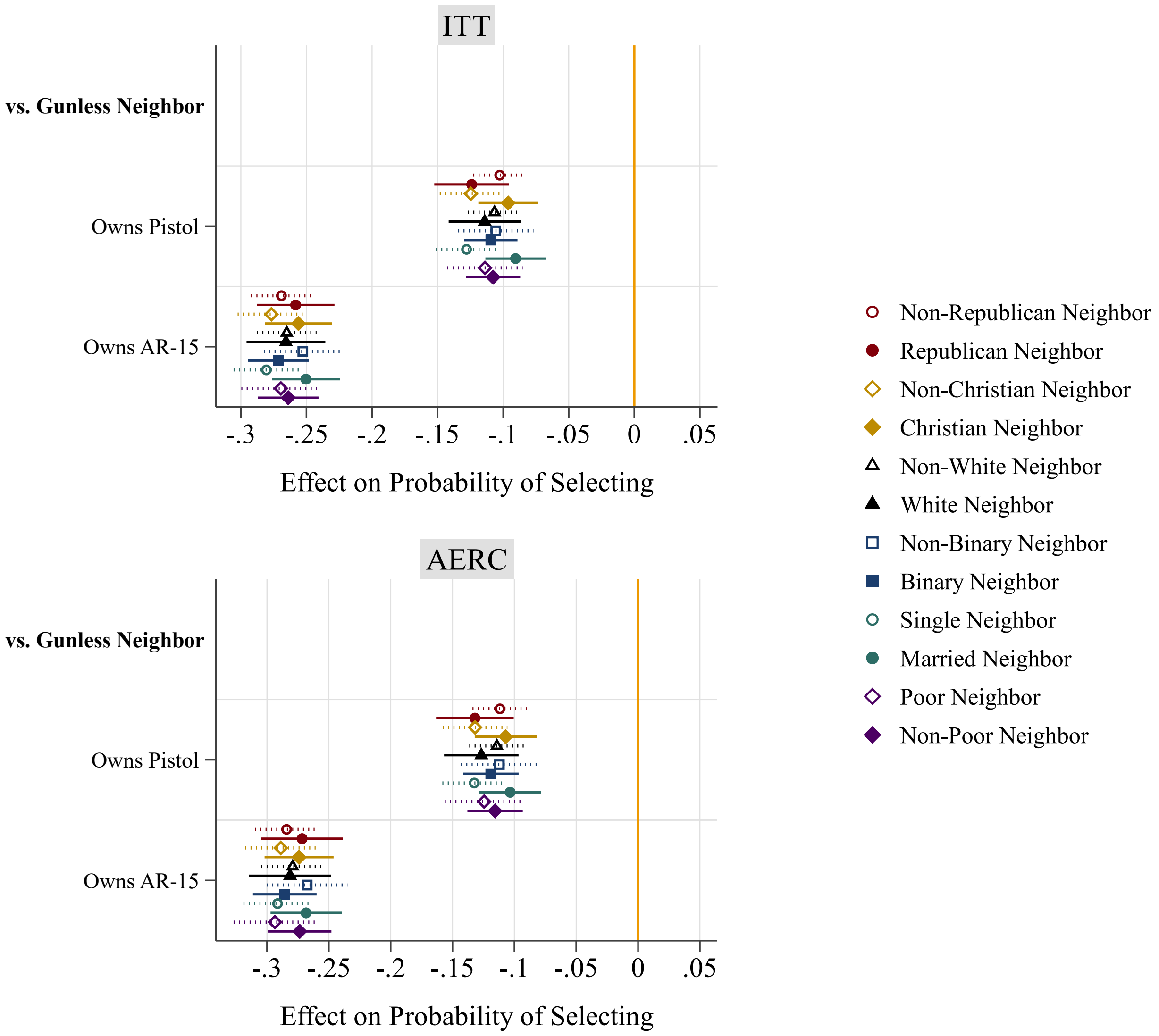

Fig. S6. Experiment 1, Low Gun Socialization Respondents: By Potential Neighbor's Attributes. This figure shows the effects of a potential neighbor's gun ownership, conditional on the neighbor's other attributes. The models are estimated using linear regression with robust standard errors clustered at the respondent level. Coefficients (with 95% confidence intervals) are shown. All models control for the other randomized attributes of the neighbor.

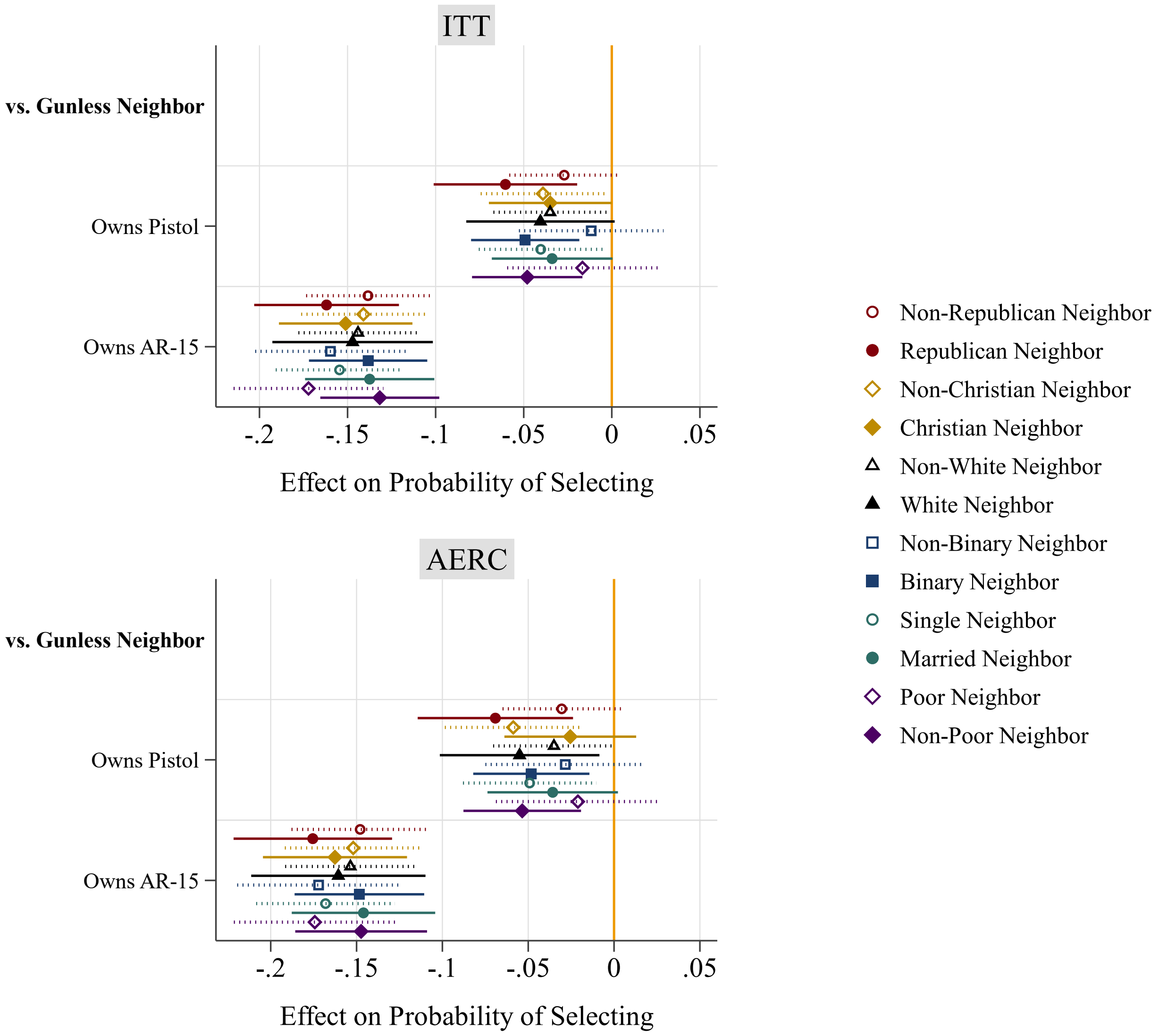

Fig. S7. Experiment 1, High Gun Socialization Respondents: By Potential Neighbor's Attributes. This figure shows the effects of a potential neighbor's gun ownership, conditional on the neighbor's other attributes. The models are estimated using linear regression with robust standard errors clustered at the respondent level. Coefficients (with 95% confidence intervals) are shown. All models control for the other randomized attributes of the neighbor.

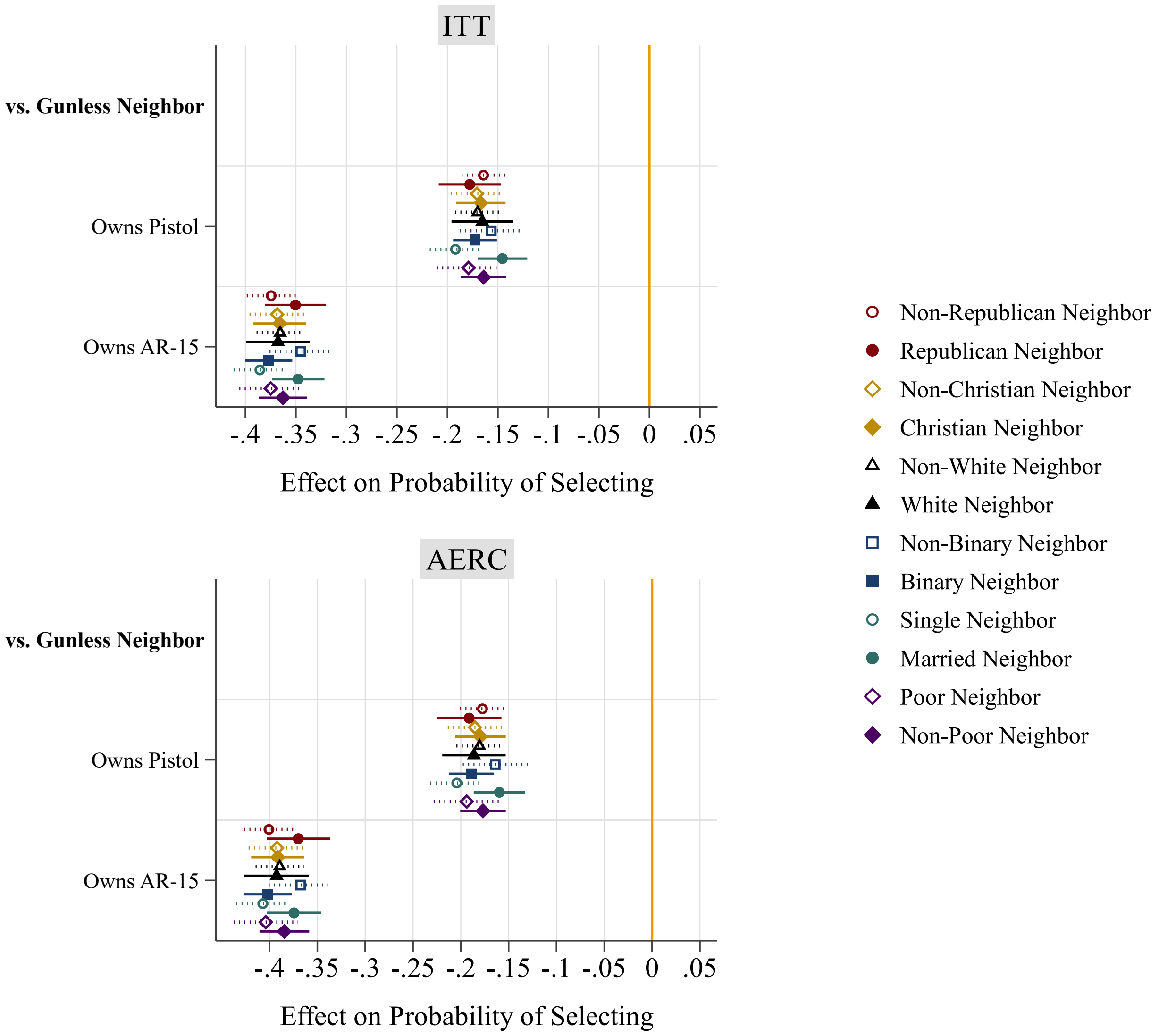

Fig. S8. Experiment 1, Low Gun Desirability Respondents: By Potential Neighbor's Attributes. This figure shows the effects of a potential neighbor's gun ownership, conditional on the neighbor's other attributes. The models are estimated using linear regression with robust standard errors clustered at the respondent level. Coefficients (with 95% confidence intervals) are shown. All models control for the other randomized attributes of the neighbor.

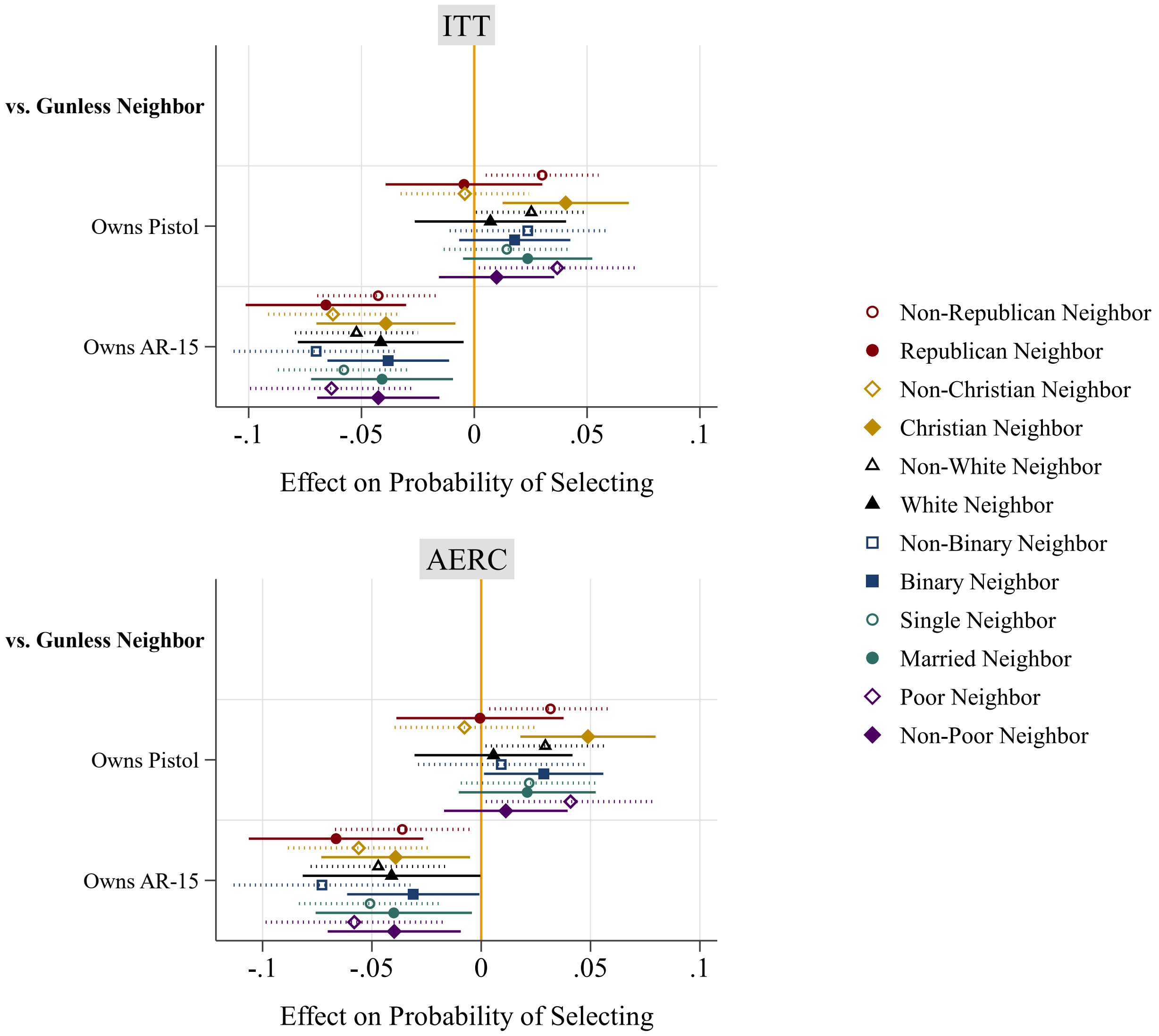

Fig. S9. Experiment 1, High Gun Desirability Respondents: By Potential Neighbor's Attributes. This figure shows the effects of a potential neighbor's gun ownership, conditional on the neighbor's other attributes. The models are estimated using linear regression with robust standard errors clustered at the respondent level. Coefficients (with 95% confidence intervals) are shown. All models control for the other randomized attributes of the neighbor.

Second Experiment:

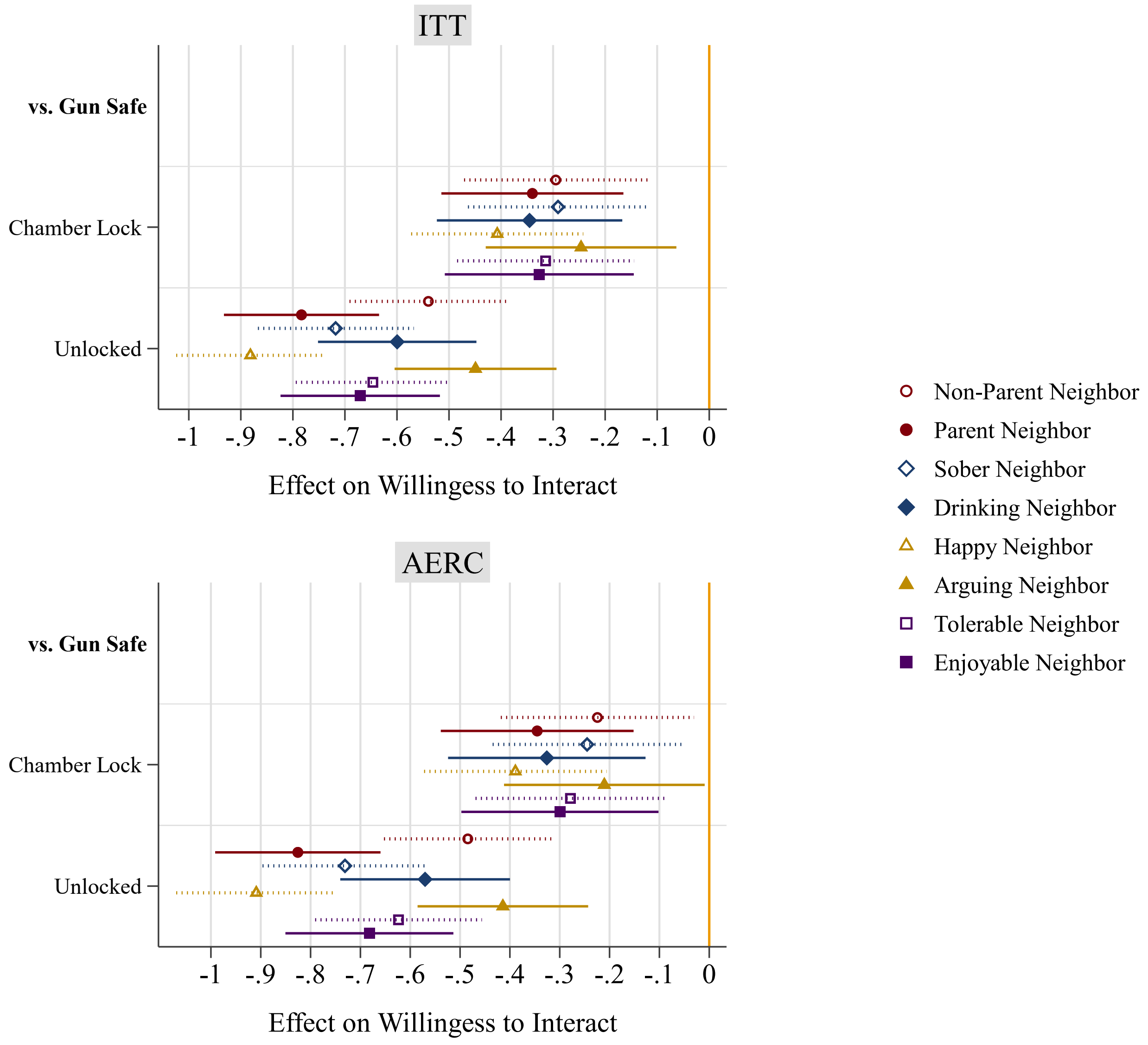

Fig. S10. Experiment 2, All Respondents: By Potential Neighbor's Attributes. This figure shows the effects of a potential neighbor's gun storage, conditional on the neighbor's other attributes. The models are estimated using linear regression with robust standard errors. Coefficients (with 95% confidence intervals) are shown. All models control for the other randomized attributes of the neighbor.

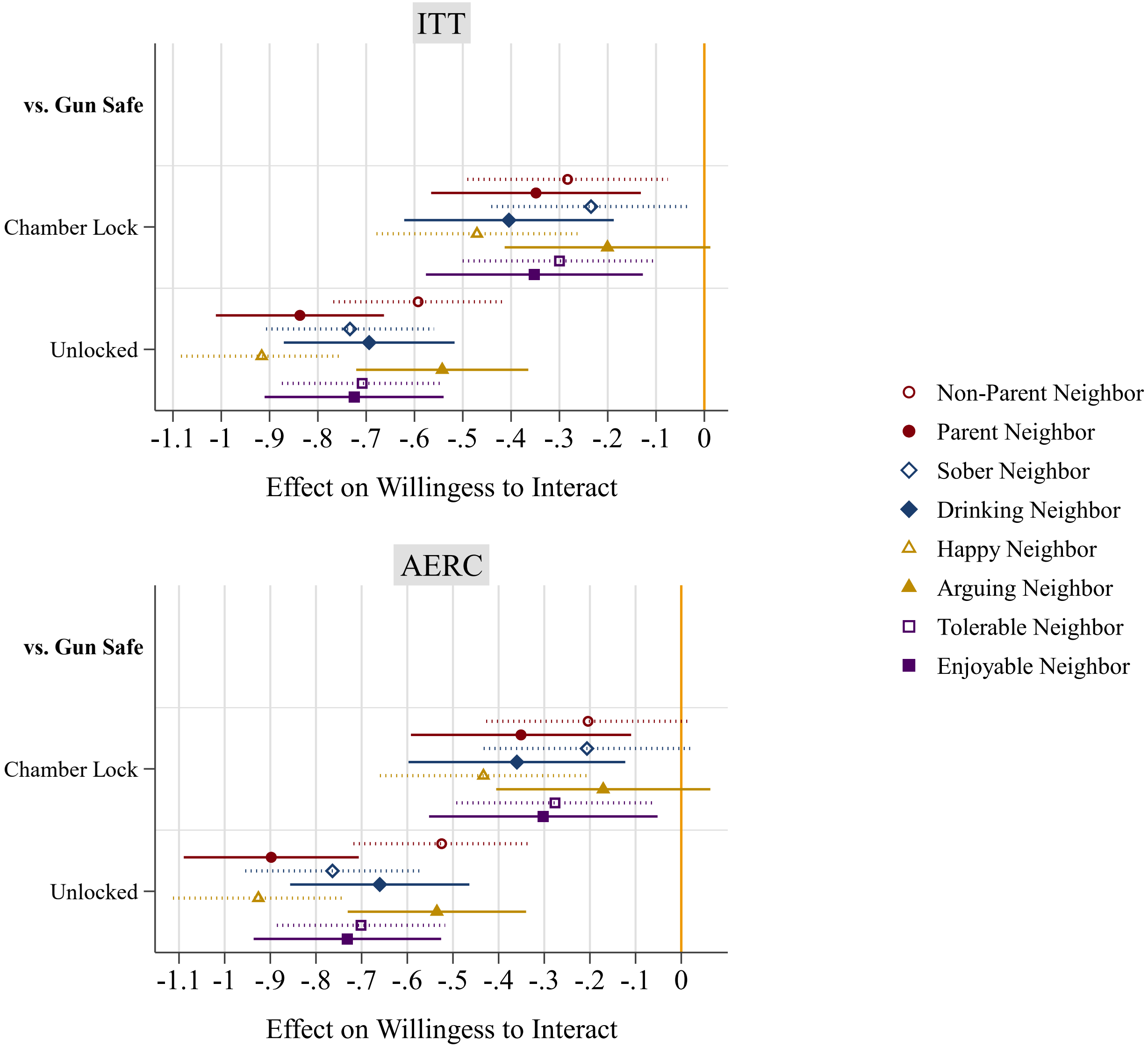

Fig. S11. Experiment 2, Non-Republican Respondents: By Potential Neighbor's Attributes. This figure shows the effects of a potential neighbor's gun storage, conditional on the neighbor's other attributes. The models are estimated using linear regression with robust standard errors. Coefficients (with 95% confidence intervals) are shown. All models control for the other randomized attributes of the neighbor.

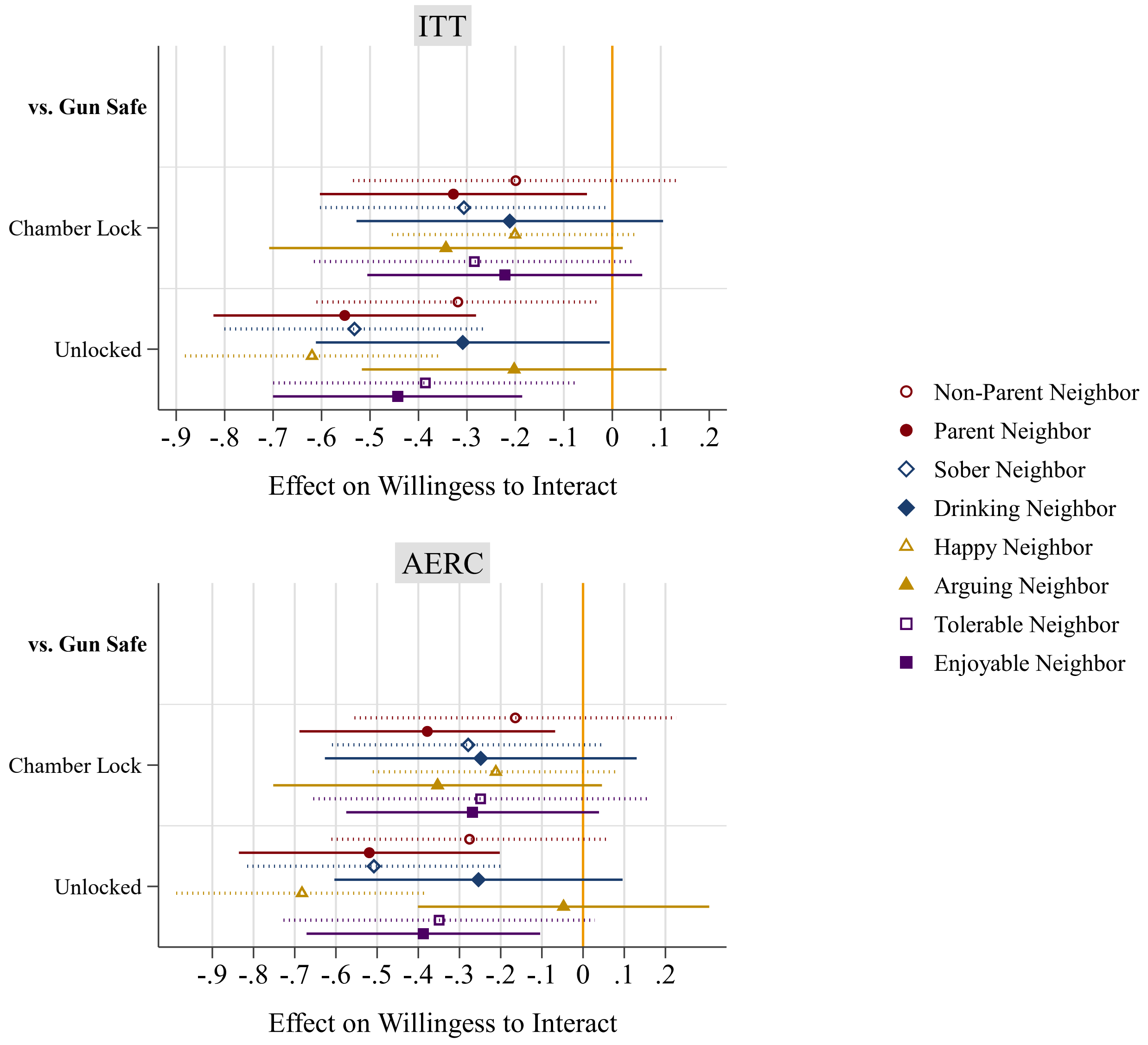

Fig. S12. Experiment 2, Republican Respondents: By Potential Neighbor's Attributes. This figure shows the effects of a potential neighbor's gun storage, conditional on the neighbor's other attributes. The models are estimated using linear regression with robust standard errors. Coefficients (with 95% confidence intervals) are shown. All models control for the other randomized attributes of the neighbor.

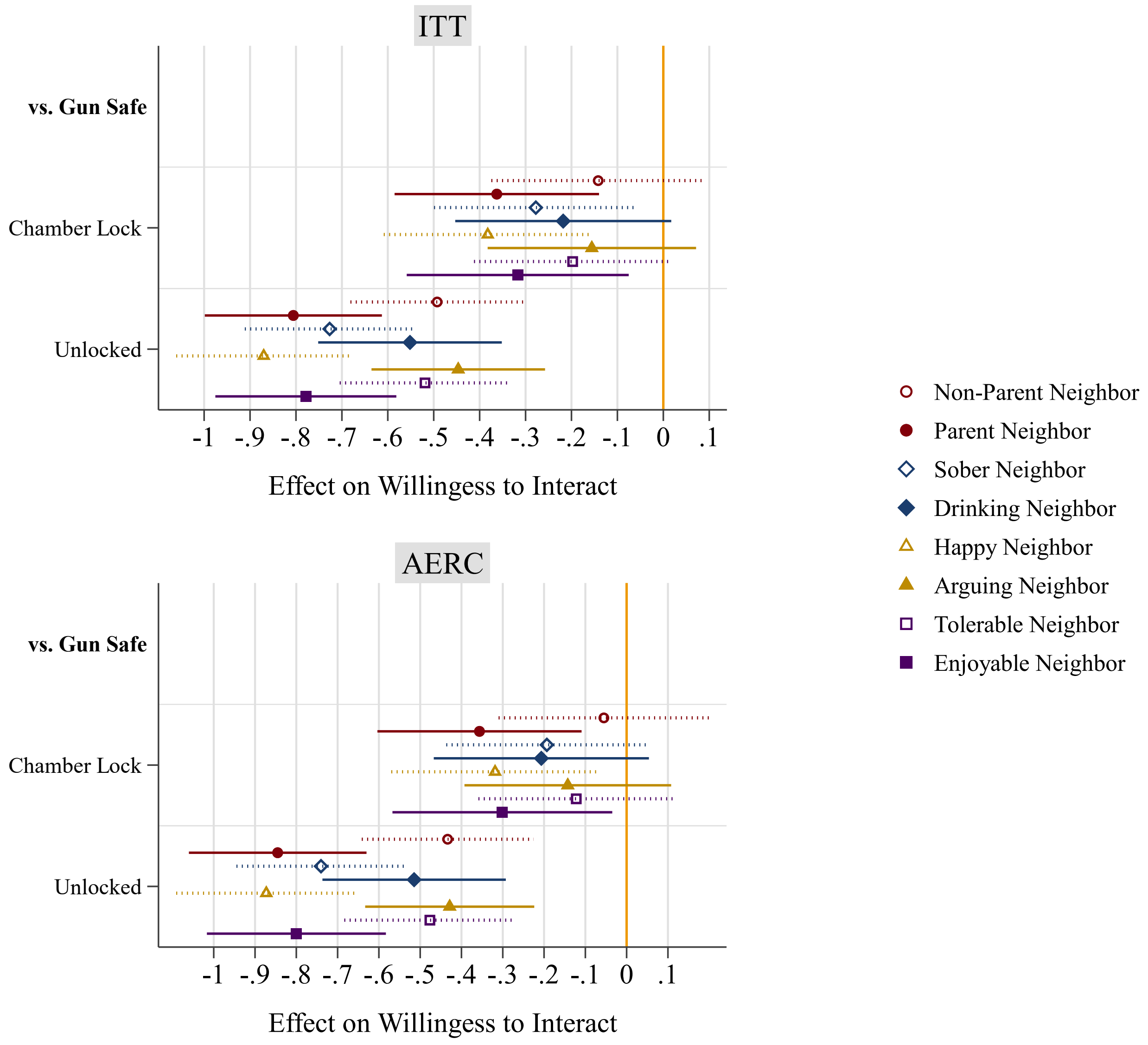

Fig. S13. Experiment 2, Non-Owner Respondents: By Potential Neighbor's Attributes. This figure shows the effects of a potential neighbor's gun storage, conditional on the neighbor's other attributes. The models are estimated using linear regression with robust standard errors. Coefficients (with 95% confidence intervals) are shown. All models control for the other randomized attributes of the neighbor.

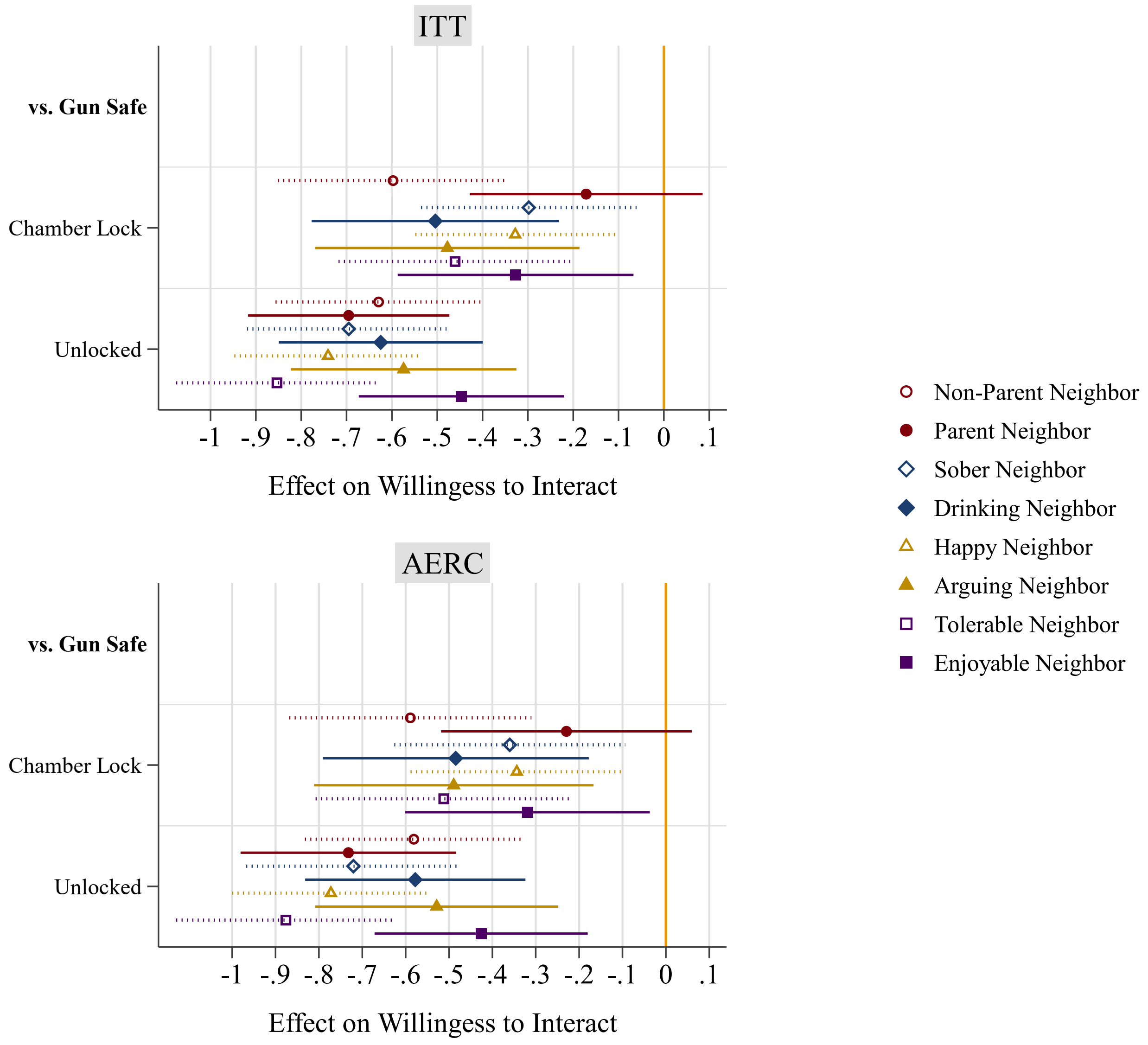

Fig. S14. Experiment 2, Owner Respondents: By Potential Neighbor's Attributes. This figure shows the effects of a potential neighbor's gun storage, conditional on the neighbor's other attributes. The models are estimated using linear regression with robust standard errors. Coefficients (with 95% confidence intervals) are shown. All models control for the other randomized attributes of the neighbor.

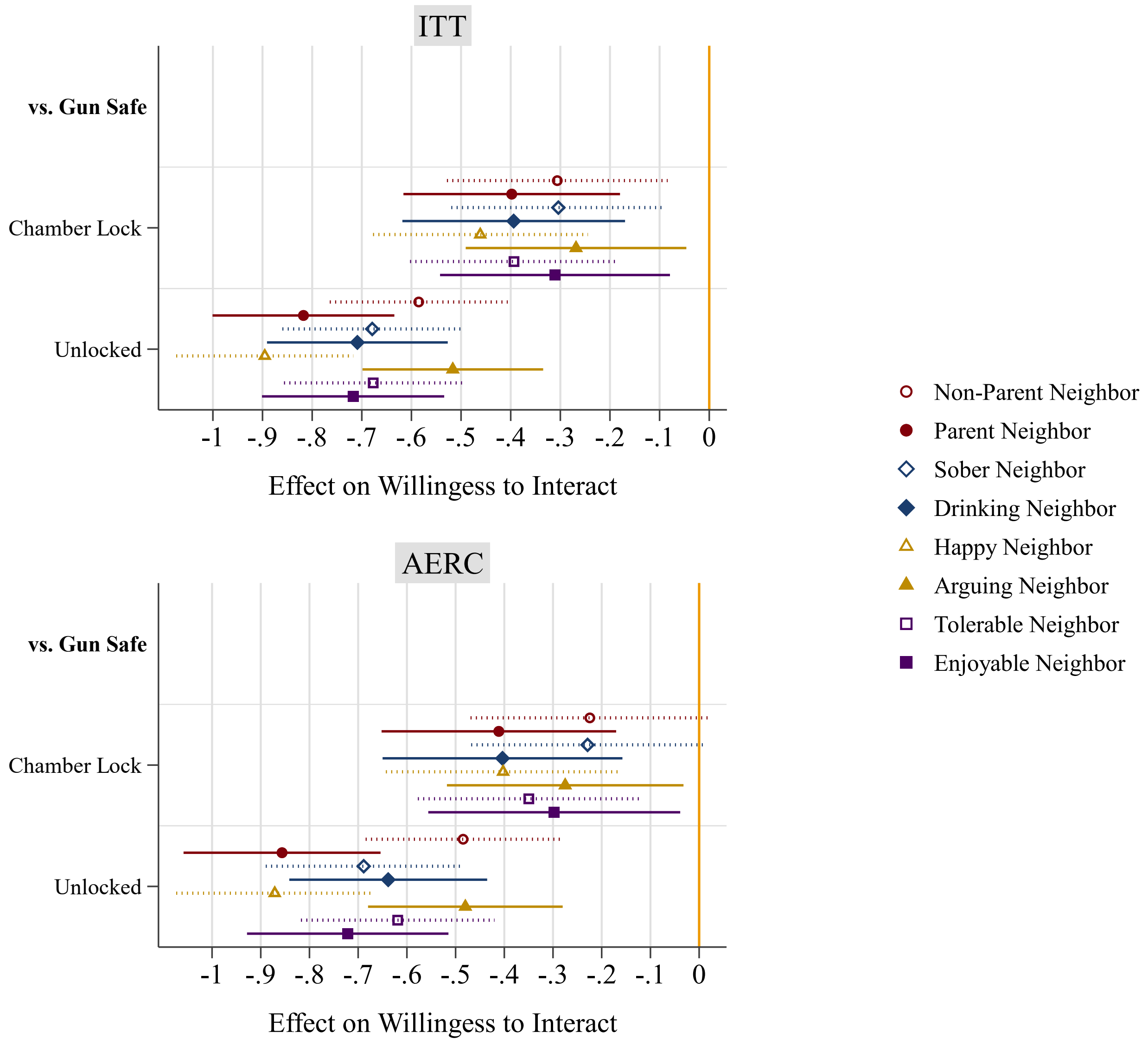

Fig. S15. Experiment 2, Low Gun Socialization Respondents: By Potential Neighbor's Attributes. This figure shows the effects of a potential neighbor's gun storage, conditional on the neighbor's other attributes. The models are estimated using linear regression with robust standard errors. Coefficients (with 95% confidence intervals) are shown. All models control for the other randomized attributes of the neighbor.

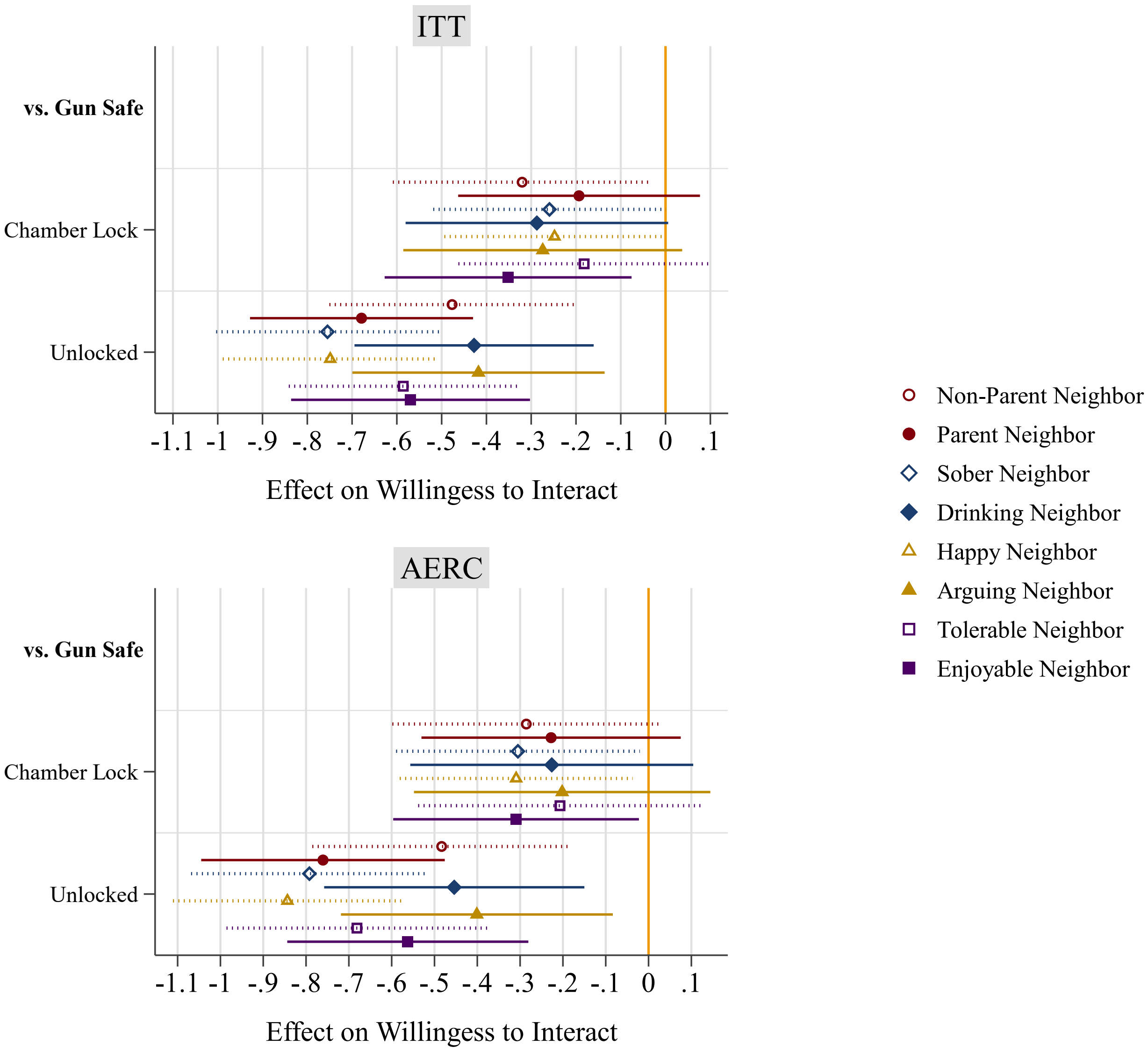

Fig. S16. Experiment 2, High Gun Socialization Respondents: By Potential Neighbor's Attributes. This figure shows the effects of a potential neighbor's gun storage, conditional on the neighbor's other attributes. The models are estimated using linear regression with robust standard errors. Coefficients (with 95% confidence intervals) are shown. All models control for the other randomized attributes of the neighbor.

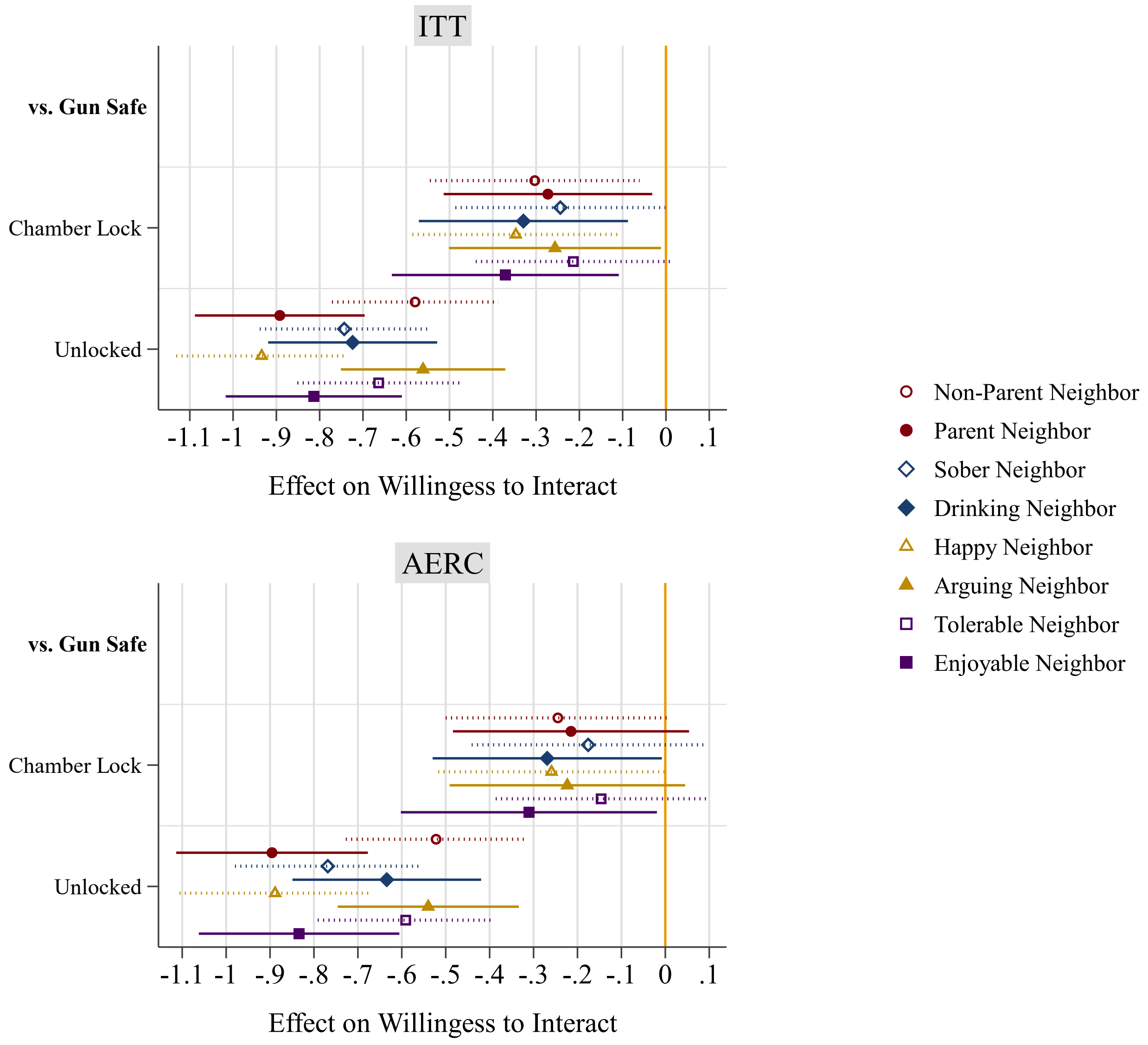

Fig. S17. Experiment 2, Low Gun Desirability Respondents: By Potential Neighbor's Attributes. This figure shows the effects of a potential neighbor's gun storage, conditional on the neighbor's other attributes. The models are estimated using linear regression with robust standard errors. Coefficients (with 95% confidence intervals) are shown. All models control for the other randomized attributes of the neighbor.

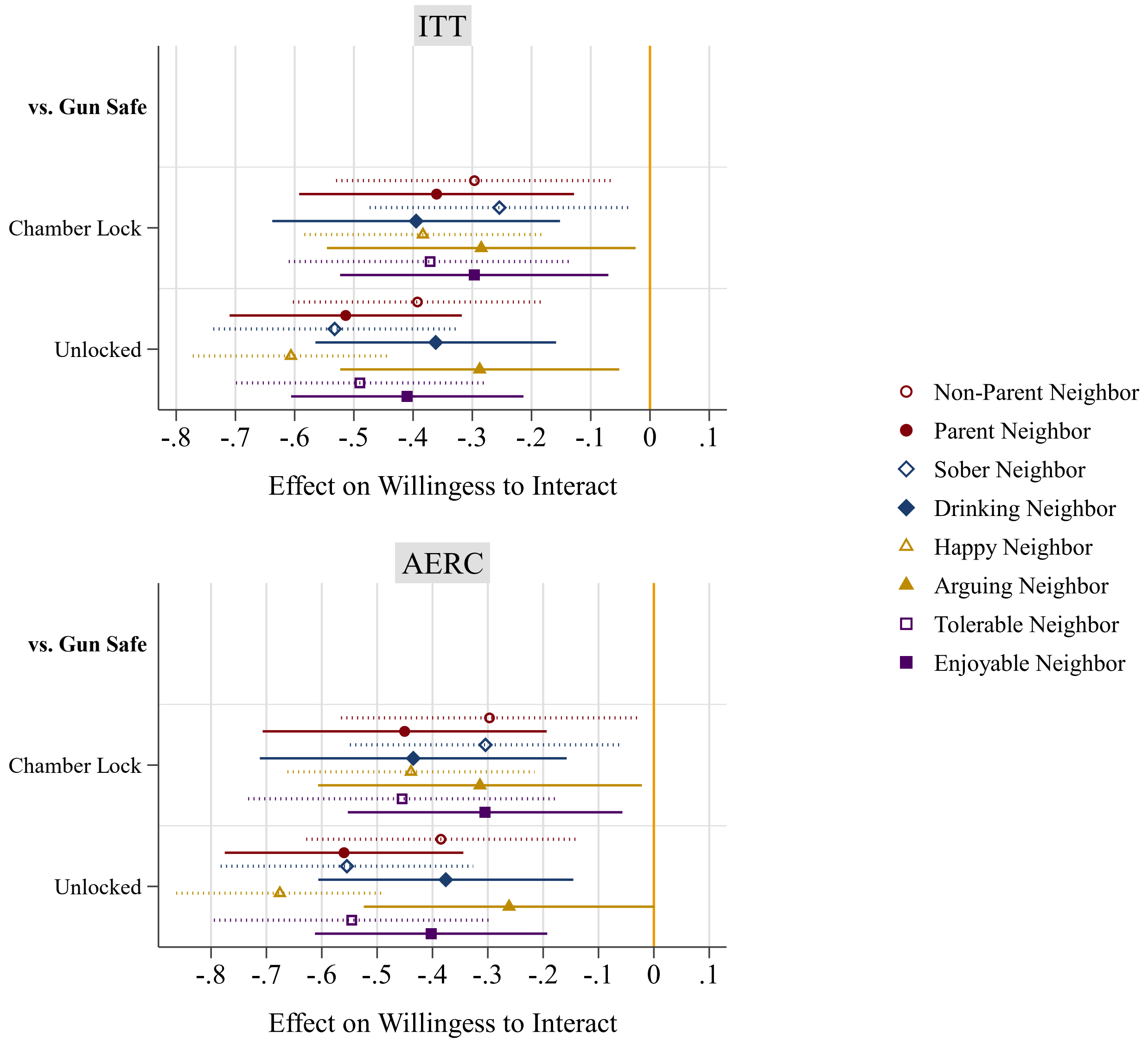

Fig. S18. Experiment 2, High Gun Desirability Respondents: By Potential Neighbor's Attributes. This figure shows the effects of a potential neighbor's gun storage, conditional on the neighbor's other attributes. The models are estimated using linear regression with robust standard errors. Coefficients (with 95% confidence intervals) are shown. All models control for the other randomized attributes of the neighbor.

ANALYSES USING SAMPLING WEIGHTS

➢ NOTE: These supplementary analyses re-estimate the main models after applying sampling weights (generated via raking on marginal distributions, see Table S2).

First Experiment:

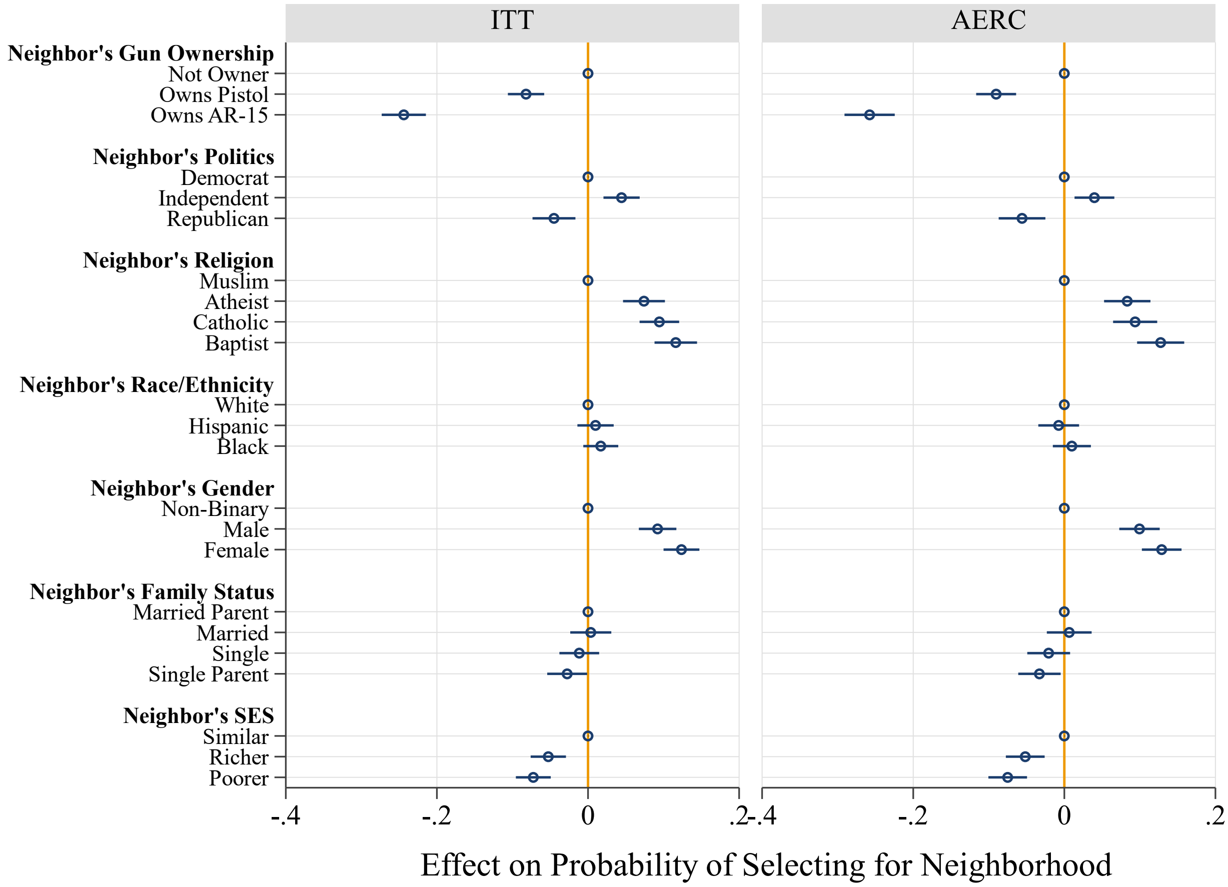

Figure S19. Experiment 1: Weighted. Models are estimated using linear regression with robust standard errors clustered at the respondent level. Coefficients (with 95% confidence intervals) are shown.

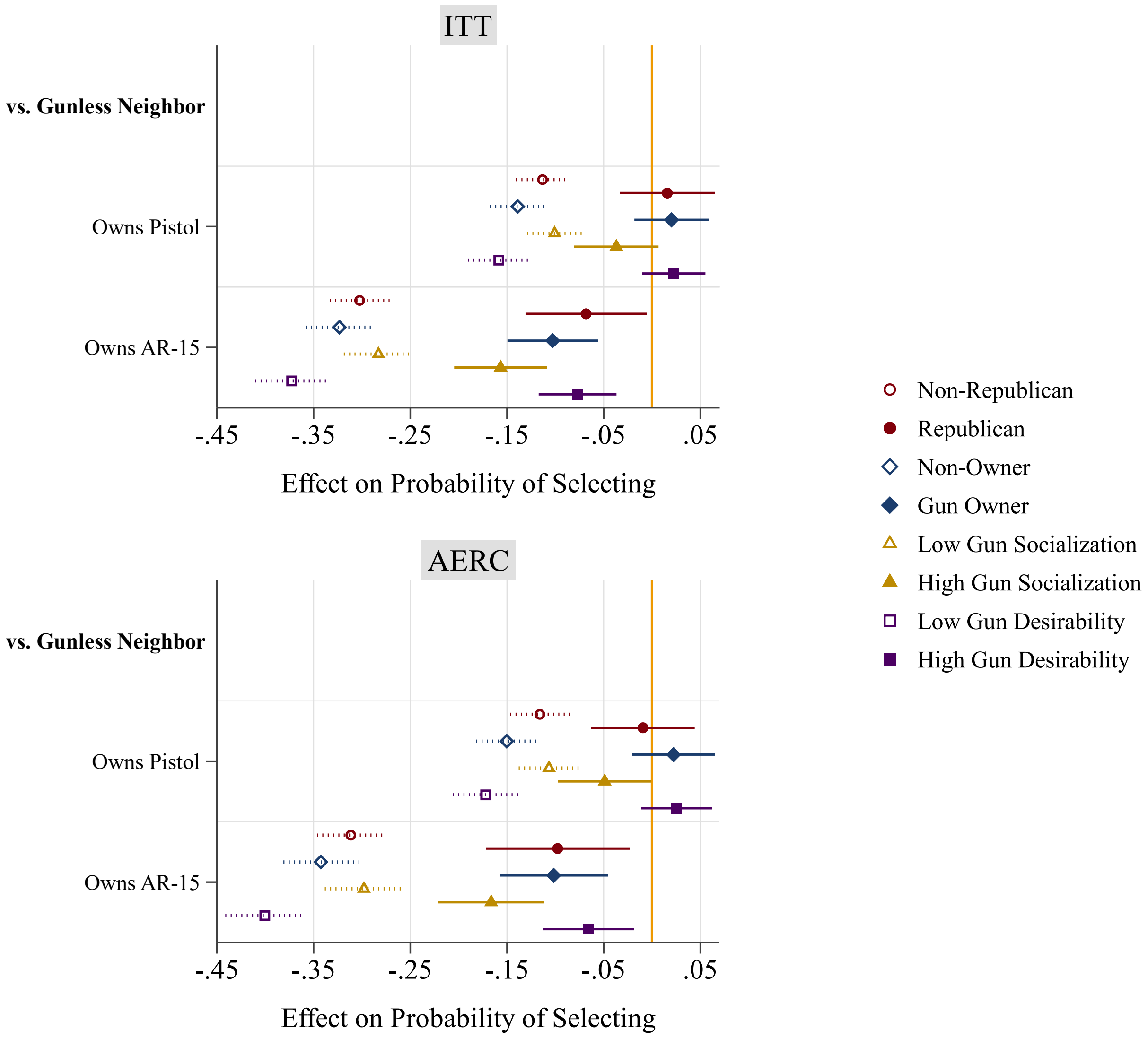

Figure S20. Experiment 1, Disaggregated Analyses: Weighted. Models are estimated using linear regression with robust standard errors clustered at the respondent level, and control for the six other randomized attributes of the applicant. Regression coefficients (with 95% confidence intervals) are shown. “Low” is defined as at or below the mean on the variable, and “High” is above the mean.

Second Experiment:

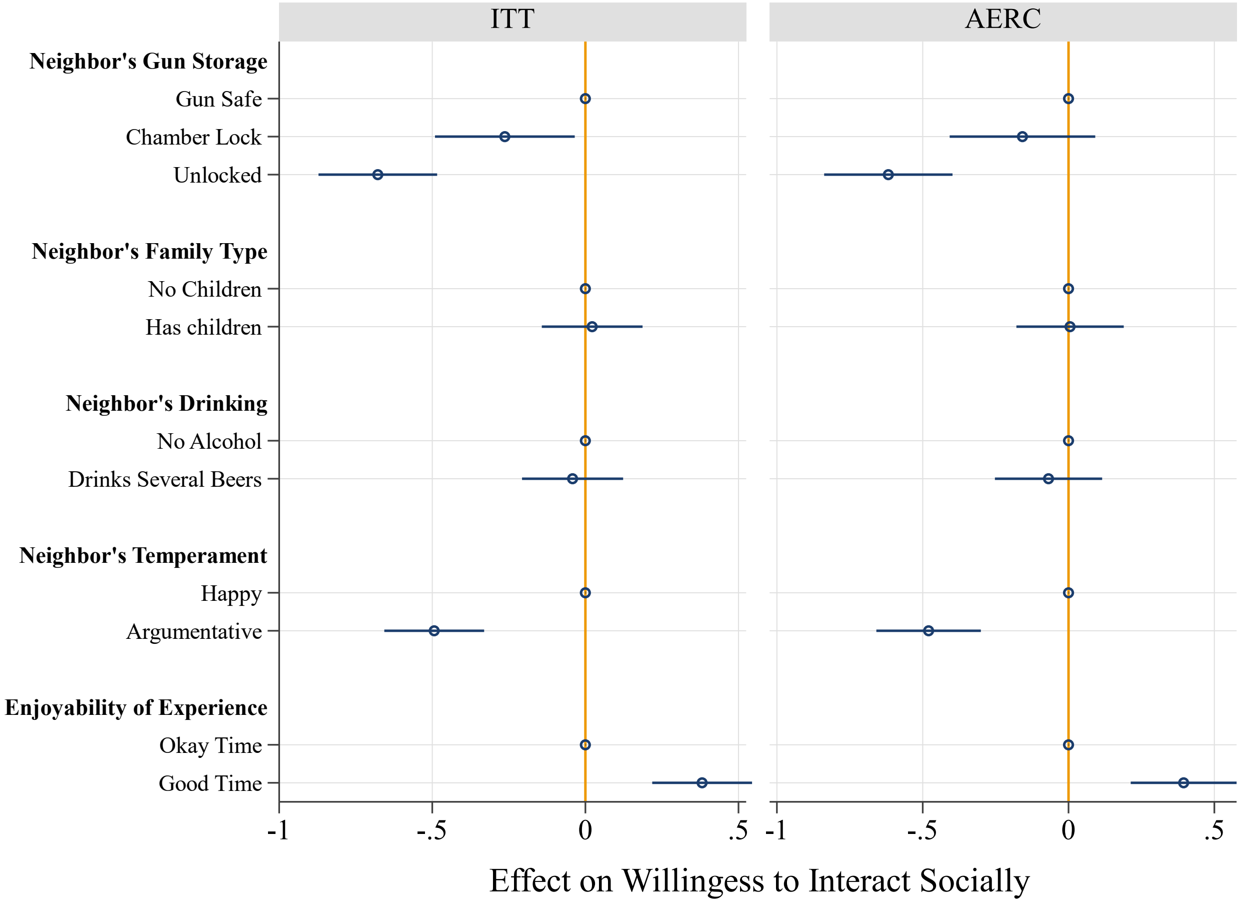

Figure S21. Experiment 2: Weighted. Models are estimated using linear regression with robust standard errors. Coefficients (with 95% confidence intervals) are shown. ITT = intent-to-treat effect, AERC = average effect of receipt of treatment for compliers.

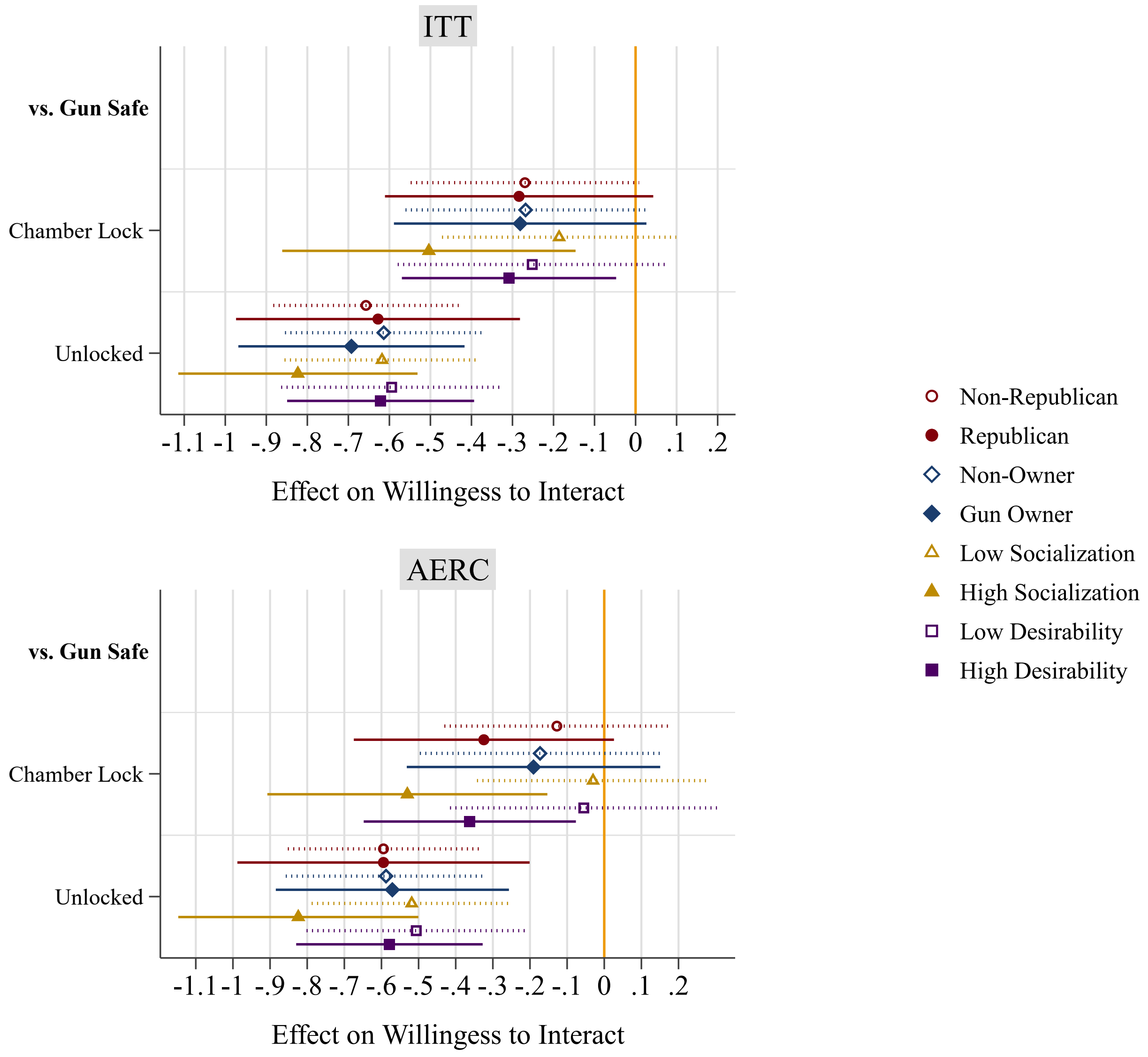

Figure S22. Experiment 2, Disaggregated Analyses: Weighted. Models are estimated using linear regression with robust standard errors. Coefficients (with 95% confidence intervals) are shown. "Low" is defined as at or below the mean on the variable, and "High" is above the mean.

Table S2. Descriptive Statistics

Variables	Unweighted Sample	Weighted Sample	18+ Population
Region			
Northeast	18.9%	17.5%	17.5%[A]
Midwest	21.4%	20.6%	20.6%[A]
South	41.7%	38.4%	38.4%[A]
West	18.0%	23.6%	23.6%[A]
Sex			
Male	46.2%	49.2%	49.2%[A]
Female	53.8%	50.8%	50.8%[A]
Race			
White alone	79.6%	76.8%	76.9%[A]
Black alone	8.9%	13.1%	13.1%[A]
Asian alone	5.1%	6.04%	6.4%[A]
Other alone	2.3%	1.5%	1.5%[A]
Two or more races	4.1%	2.2%	2.2%[A]
Ethnicity			
Not Hispanic	90.1%	82.8%	82.8%[A]
Hispanic	9.9%	17.2%	17.2%[A]
Age			
18 to 24	6.0%	12.0%	12.0%[A]
25 to 44	58.1%	34.2%	34.2%[A]
45 to 64	30.5%	31.6%	31.6%[A]
65+ years	5.4%	22.2%	22.2%[A]
Education			
High school degree or less	12.7%	37.7%	37.7%[B]
Some college	31.1%	29.3%	29.3%[B]
Bachelor's degree or higher	56.2%	33.0%	33.0%[B]
Household Income			
Under $20,000	9.6%	13.3%	13.3%[C]
$20,000 to $59,999	36.2%	31.1%	31.1%[C]
$60,000 or more	54.2%	55.6%	55.5%[C]
Partisan Identification			
Democrat	37.7%	29.3%	29.3%[C]
Independent	35.0%	45.9%	45.9%[C]
Republican	27.3%	24.8%	24.8%[C]
Marital Status			
Not married	56.1%	50.0%	50.0%[C]
Married	43.9%	50.0%	50.0%[C]
Household Gun Ownership			
No	65.1%	64.3%	64.3%[C]
Yes	34.9%	35.7%	35.7%[C]

NOTES: [A]2022 U.S. Census estimates; [B]2022 American Community Survey 1-Year Estimates; [C]2022 General Social Survey. The weights were constructed via iterative proportional fitting.
